# Quantitative diagnosis of rotator cuff tears based on sonographic pattern recognition

**DOI:** 10.1371/journal.pone.0212741

**Published:** 2019-02-28

**Authors:** Ruey-Feng Chang, Chung-Chien Lee, Chung-Ming Lo

**Affiliations:** 1 Graduate Institute of Biomedical Electronics and Bioinformatics, National Taiwan University, Taipei, Taiwan; 2 Department of Computer Science and Information Engineering, National Taiwan University, Taipei, Taiwan; 3 Department of Orthopedic Surgery, New Taipei City Hospital, New Taipei City, Taiwan; 4 Department of Orthopedic Surgery, National Taiwan University Hospital, Taipei, Taiwan; 5 Graduate Institute of Biomedical Informatics, College of Medical Science and Technology, Taipei Medical University, Taipei, Taiwan; 6 Graduate Institute of Library, Information and Archival Studies, National Chengchi University, Taipei, Taiwan; New York Chiropractic College, UNITED STATES

## Abstract

The lifetime prevalence of shoulder pain is nearly 70% and is mostly attributable to subacromial disorders. A rotator cuff tear is the most severe form of subacromial disorders, and most occur in the supraspinatus. For clinical examination, shoulder ultrasound is recommended to detect supraspinatus tears. In this study, a computer-aided tear classification (CTC) system was developed to identify supraspinatus tears in ultrasound examinations and reduce inter-operator variability. The observed cases included 89 ultrasound images of supraspinatus tendinopathy and 102 of supraspinatus tear from 136 patients. For each case, intensity and texture features were extracted from the entire lesion and combined in a binary logistic regression classifier for lesion classification. The proposed CTC system achieved an accuracy rate of 92% (176/191) and an area under receiver operating characteristic curve (Az) of 0.9694. Based on its diagnostic performance, the CTC system has promise for clinical use.

## Introduction

Rotator cuff disorders are the most common cause (up to 70%) of shoulder pain [1], with a lifetime prevalence approaching 70% [2]. The financial burden of shoulder pain on the United States health care system is estimated at $7 billion annually [3], and the substantial loss of productivity is often underestimated. The mechanisms of rotator cuff diseases are believed to possess a dynamic pathology, with subacromial impingement as the initial stage and rotator cuff tear as the final stage [4]. Rotator cuff disorders include tendinopathy, calcific tendinitis, tears, bursitis, and bursal reactions [5]. Among these disorders, rotator cuff tears, which have a prevalence rate of 20.7%, are the most severe forms [6]. Individuals suffering from rotator cuff tears may have severe shoulder pain, weak forward elevation, abduction or external rotation, which can detrimentally affect the activities of daily life.

The accurate diagnosis of rotator cuff disorders is important to determine treatment strategy, especially differentiating tears from other types of tendinopathies [7, 8]. The presence of rotator cuff full-thickness tears influence the decision to undertake the surgical repair or arthroplasty [9]. Furthermore, the measurement of size and location of rotator cuff tear is necessary for pre-operative planning. Clinically, imaging examinations such as shoulder ultrasound, roentgenogram, magnetic resonance imaging (MRI), and magnetic resonance arthrography (MRA) are necessary for assessing rotator cuff tears because physical examinations and clinical symptoms may be unreliable [10, 11]. Compared to other imaging modalities, shoulder ultrasound has the advantages of being inexpensive, conducted in real-time, and convenient to operate. Nevertheless, using ultrasound is operator dependent which relies on adequate training and experience in diagnosing rotator cuff tears. Especially, differentiating partial and full-thickness tears from other tendinopathies is a challenge [12]. According to previous literature [13–19], the diagnostic sensitivity and specificity of shoulder ultrasound on tear detection has a range of 46%-95% and 50%-95%, respectively. The variabilities are highly correlated with the level of experience of the operator and the patterns of the rotator cuff tears [20]. Experienced musculoskeletal radiologists or shoulder orthopedic surgeons possess a higher accuracy than general radiologists and ultrasonographers in diagnosing rotator cuff tears via shoulder ultrasound [20]. From this viewpoint, the inter-observer variability in diagnosing rotator cuff tears between operators with different professions or levels of experience is substantial, and higher variability is demonstrated in the diagnosis of partial-thickness tears [21, 22]. The introduction of quantitative and automated diagnostic procedures could potentially reduce the impact of variability.

Computer-aided diagnosis systems provide an objective, quantitative assessment of lesion type and grade [23–26]. After defining the lesion area with manual or semi-automatic segmentation, quantitative features can be extracted and combined in an artificial intelligence classifier. By considering a broad range of relevant features, sonographic patterns such as echogenicity and textures are modeled and used to recognize incoming cases [27]. A previous study demonstrated that the likelihood estimation of a computer-aided diagnosis system can be used to reduce observer variability [28]. In this research, consistently high performance in the differentiation of breast tumors was achieved with the assistance of a computer-aided diagnosis system. For residents, the specificity of the breast tumor diagnosis was improved from 20% to 40% (*p*-value < 0.01) and the κ value from 0.09 to 0.53 (*p*-value < 0.001). For dedicated breast imagers, the specificity was increased from 34% to 43% (*p*-value = 0.16) and the κ value from 0.21 to 0.61 (*p*-value < 0.001).

In this study, a computer-aided tear classification (CTC) system based on the quantitative intensity and texture features was proposed to classify rotator cuff tears in shoulder ultrasounds. The establishment of the CTC system is expected to provide consistent and objective recommendations to junior physicians for clinical examinations.

## Materials and methods

### Patients and data acquisition

This study was approved by the Institutional Review Board of New Taipei City Hospital, and informed consent was waived. Between January 2012 and February 2016, patients attending the orthopedic department in New Taipei City Hospital with shoulder symptoms who underwent shoulder ultrasound examination were selected to this study. All these patients followed the indication of shoulder ultrasound examination. All shoulders were imaged using an ALOKA alpha-6 ultrasound scanner (Hitachi-Aloka Medical, Tokyo, Japan) with a linear array probe (scan width: 36 mm) ranging from 5 to 13 MHz by an orthopedic shoulder surgeon who is also specialized in musculoskeletal ultrasound. The acquisition frequency was 8 MHz and depth of scanning was 4 cm with the focus of supraspinatus layer. All patients selected to this study underwent only drug control or other conservative treatment before ultrasound examination. The patients with post-operative intervention and recent injection including hyaluronic acid, steroid and platelet-rich plasma (PRP) injection were excluded. The patient population (n = 136) included 61 males and 75 females between 25 and 86 years of age, with a mean age of 58.7 years, and 32 of them underwent bilateral shoulder ultrasound evaluation due to bilateral shoulder symptoms. In 23 shoulders, because the shoulder morphology appeared non-uniform, two ultrasound images of different long-axis cut were captured from a shoulder. The image database was composed of 191 shoulder images including 89 images of supraspinatus tendinopathy and 102 images of supraspinatus tear. Forty-two of 102 supraspinatus tears were full-thickness tears. Image selection and diagnosis of 191 shoulder ultrasound images were all confirmed by an orthopedic shoulder surgeon and a physical medicine and rehabilitation (PM&R) physician who was specialized in shoulder musculoskeletal ultrasound.

**Fig 1:**
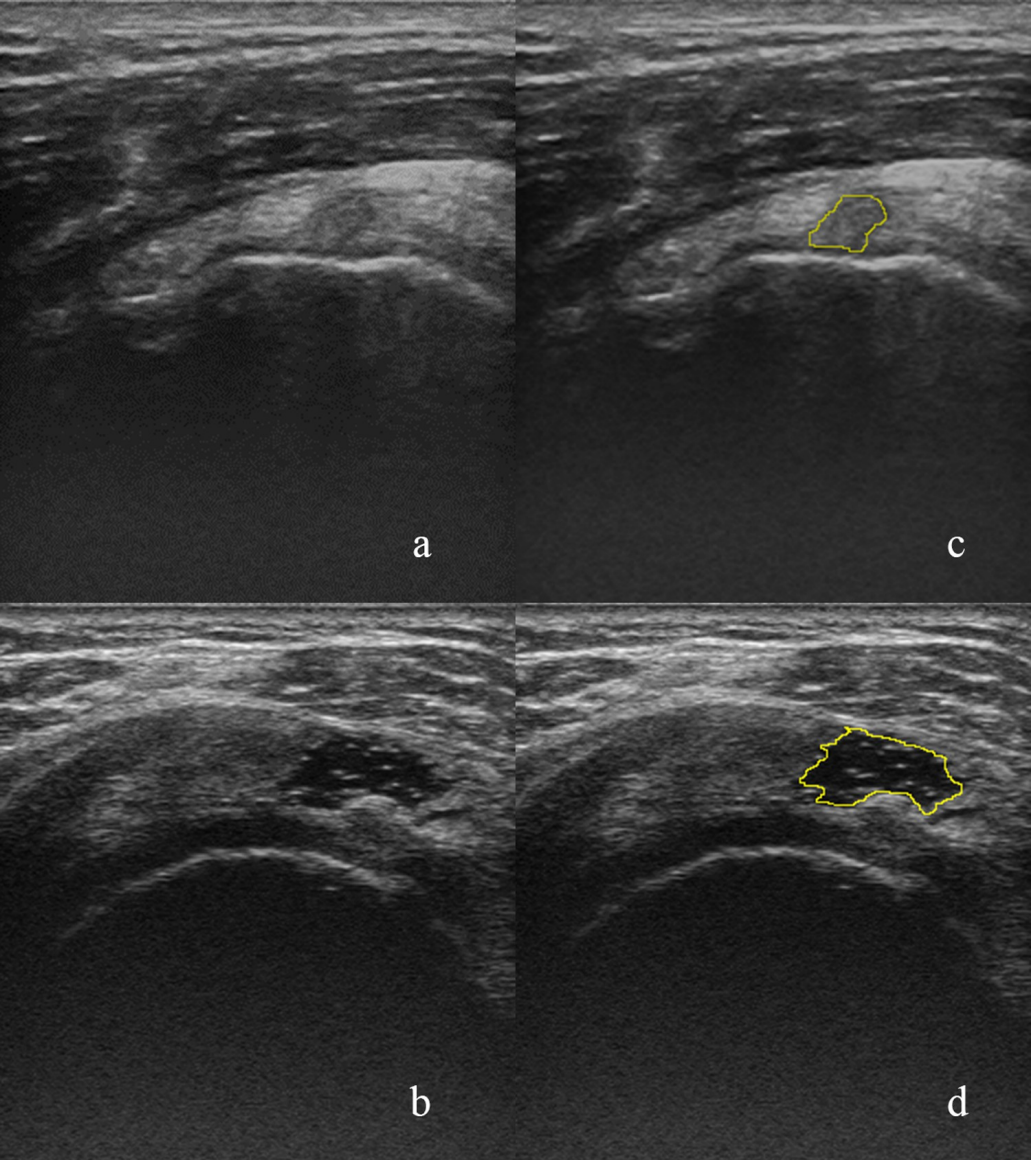
Supraspinatus tendon shown in ultrasound images. (a) A case of tendon tendinopathy. (b) A case of supraspinatus tear. (c) and (d): The lesion contours of (a) and (b), respectively, which were delineated by a shoulder orthopedic surgeon using ImageJ.

During the ultrasound examination, patients were placed in a standard sitting position with shoulder extended, internally rotated, and a routine ultrasound procedures were followed. The settings of the ultrasound scanner, such as time gain compensation, were consistent for all patients. The acquired shoulder ultrasound images were stored as 8-bit images with gray-scale values ranging from 0 to 255. According to previous meta-analysis study, diagnostic accuracy of supraspinatus tears is high while performed by musculoskeletal radiologists and shoulder orthopedic surgeons [20]. As the gold standard in the evaluation of the proposed CTC system, an orthopedic shoulder surgeon and a PM&R physician who was specialize in shoulder musculoskeletal ultrasound classified the lesions into 89 cases of supraspinatus tendon tendinopathy and 102 cases of supraspinatus tear. Lesion areas were delineated by the same orthopedic shoulder surgeon to enclose the necessary tissues while avoiding normal tendons. Image J was the software used in showing ultrasound images and delineating. The delineation of supraspinatus lesion areas was also confirmed by the PM&R physician to obtain the consensus. Fig 1 provides ultrasound images of a case of tendinopathy and a case of a tear.

### Feature extraction

The normal supraspinatus tendon is a convex beak-shaped hyperechoic structure in long-axis view [29]. After the delineation of the lesion area in the supraspinatus tendon, the sonographic appearance of the enclosed tissues was analyzed according to their echogenic properties. Supraspinatus tendinopathies were irregular, and loss of homogeneous texture was observed. Supraspinatus tears appeared to have irregular margins with hypoechoic areas [30, 31]. These tears, particularly those associated with tendon thickness, can be full-thickness tears or partial-thickness tears from the bursal to the articular surface [32]. Consequently, intensity and texture features were proposed to analyze the tissues enclosed in the delineated lesion area.

#### Intensity features

The gray-scale distribution of tissues in the lesion can be presented by a probability distribution and form a histogram. The statistical characteristics of the histogram can be quantified by the histogram moments [33, 34]. The quantitative moments provide objective measures of the histogram, expressing the intensity difference between tendinopathies and tears. These include the mean, variance, skewness, and kurtosis, namely, the first-, second-, third-, and fourth-order central moments of a histogram. The mean, at the center of a distribution, can be obtained by summarizing total pixel values and dividing the sum by the pixel number. Variance indicates how uniform the gray-scale values are spread out. Skewness estimates the symmetry of the value distribution such as a bias to one side or not. Taking normal distribution as a reference, kurtosis is a single-peaked shape with heavily weighted tails.

#### Texture features

Another category of quantitative features proposed in computer-aided diagnosis systems for tissue characterization is texture feature [24], as tendinopathy generally appears to have heterogeneous patterns and tears appear to have hypoechoic echogenicities. The gray-scale intensities of echogenicities and statistical correlation between pixel values would provide useful information for differentiating lesion types. In this study, the gray-scale co-occurrence matrices (GLCM) [35, 36], which calculated the second-order statistics of ultrasound texture inside the lesion area, were proposed as features. The statistics revealed the correlations between adjacent pixels with different combinations of gray-scales.

**Fig 2:**
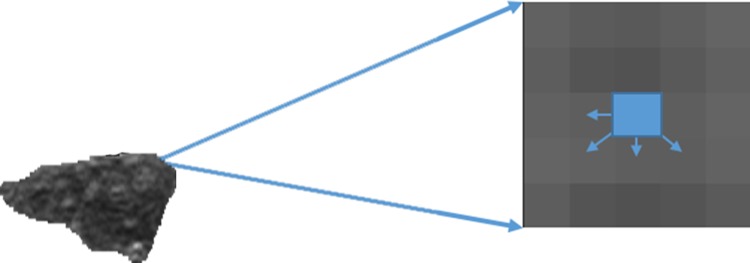
The illustration of texture analysis considering neighboring pixel pairs of four directions: 0°, 45°, 90°, and 135° and distance = 1.

Originally, the pixel values ranged from 0 to 255. These values can be separated into reduced intensity bins to achieve computational efficiency. Therefore, the first step was to quantize the original image to be the image *G* with 8 levels. For clinical diagnosis, 8 levels is enough to interpret the patterns human can distinguish. More levels would lead to unnecessary computation loading which may not suitable in clinical use. Afterward, the 8×8 co-occurrence matrices *P* = [*p*(*i*,*j*|*d*,*θ*)] were generated by scanning the pixels and their neighbors in *G*. The matrix element *P* = [*p*(*i*,*j*|*d*,*θ*)] represented the frequency of two adjacent pixels with values of *i* and *j* at a distance (*d*) and a direction (*θ*). Based on the matrix, 14 GLCM texture features were calculated [36]. Fig 2 illustrates the distance *d* = 1 and the direction *θ* = 0°, 45°, 90°, or 135° in the consideration of texture composition. *d* = 1 was used to better describe the details of some lesions having less than 0.5 cm. For four co-occurrence matrices with different angles which included all the combination of two adjacent neighbors, the means of the above statistic features were calculated and extracted from the lesion areas and were combined with intensity features in the classifier to express tissue characteristics, such as brightness, contrast, and heterogeneity.

### Statistical analysis

According to the sonographic appearance of supraspinatus tendinopathy and tear, the corresponding quantitative features were proposed for classification. In the evaluation, several test methods were used to determine whether these features can distinguish between tendinopathies and tissue tears. First, a Kolmogorov-Smirnov test [37] was used to determine if the value distribution of a feature was normal or not. Normally distributed features were then tested by Student’s t-test [37], and non-normally distributed features were evaluated by a Mann-Whitney U-test [37]. The resulting *p*-values <0.05 indicated whether a feature was statistically significant in distinguishing between the supraspinatus tendinopathy and tear. To generate a prediction model based on the combination of various quantitative features, different feature combinations were evaluated in the logistic regression classifier by stepwise backward elimination to discover the most relevant combination of features with the lowest error rate. The equation of feature combination in the logistic regression classifier is:

$$Tear\_probability=1/\left(1+\exp\left(-1\times \left(f_{1}\times C_{1}+\cdots f_{n}\times C_{n}-constant\right)\right)\right)$$ (1)

where *f*_*1*_, *f*_*n*_ are different features which multiply different *C*_*1*_, *C*_*n*_ as coefficients.

Due to the limited number of collected cases, the generalization ability of the prediction model was assessed by leave-one-out cross-validation. A case picked from *N* cases (the total number of collected cases) was used to test the model trained by the remaining *N*-1 cases. Summarizing the classification result of the *N* cases accomplished the prediction performance.

In the performance evaluation, each case was given a probability indicating the likelihood of tears. Cases with probability values ≥ 0.5 were classified as tears, and those < 0.5 were classified as tendinopathy. According to the gold standard established by an orthopedic shoulder surgeon and a PM&R physician, the following five performance indices were generated: accuracy, sensitivity, specificity, positive predictive value (PPV), and negative predictive value (NPV). The tradeoffs between the sensitivity and specificity were calculated and illustrated using a receiver operating characteristic (ROC) curve. Az, the area under the ROC curve, was analyzed using ROCKIT software (C. Metz, University of Chicago, Chicago, IL, USA). Other statistical testing methods were performed using SPSS (version 16 for Windows; SPSS, Chicago, IL, USA).

## Results

Tables 1 and 2 show whether the proposed image features, including intensity and texture features, can be significant in tear classification. As a result, four intensity and 11 texture features obtained a statistically significant *p*-value less than 0.001. After feature selection, the relevant image features were selected and combined in the classifier to generate a prediction model. Three performance results of the CTC system based on different feature sets are shown in Table 3. After backward elimination, three of four intensity features including *Mean*, *Skewness*, *Kurtosis* were selected and combined in the classifier. The intensity feature set attained an accuracy of 91%, a sensitivity of 92%, and a specificity of 91%. For texture features, *Correlation*, *Information measure of correlation*, and *Inverse difference normalized* were selected to be the most relevant according to their combination performance. The texture feature set attained an accuracy of 89%, a sensitivity of 89% and, a specificity of 89%. Benefiting from complementary advantages, the combined intensity and texture feature sets including selected *Mean*, *Kurtosis*, *Inverse difference normalized*, and *Inverse difference moment* achieved an accuracy of 92%, which is better than using intensity and texture feature sets individually.

**Table 1:** The test results of intensity features using the Mann-Whitney U-test.

Features	Tendinopathy	Tear	*p*-value
	Median	Median	
*Mean*	116.56	42.58	<0.001*
*Variance*	507.71	292.98	<0.001*
*Skewness*	0.26	1.19	<0.001*
*Kurtosis*	2.94	5.14	<0.001*

* *p*-value<0.05 indicates a statistically significant difference.

**Table 2:** The test results of texture features using student’s *t*-test (mean) or the Mann-Whitney U-test (median).

Features	Tendinopathy		Tear		*p*-value
	Mean±SD	Median	Mean±SD	Median	
*Autocorrelation*		1.496		1.132	<0.001*
*Contrast*	0.0179± 0.006		0.0115± 0.005		<0.001*
*Correlation*	0.973± 0.012		0.929± 0.029		<0.001*
*Cluster Prominence*		106.48		16.04	<0.001*
*Cluster Shade*		11.81		1.92	<0.001*
*Dissimilarity*		0.006		0.004	<0.001*
*Energy*		0.962		0.976	<0.001*
*Entropy*		0.136		0.090	<0.001*
*Homogeneity*		0.997		0.998	0.101
*Difference variance*	0.0179± 0.006		0.0115± 0.005		<0.001*
*Difference entropy*		0.028		0.025	0.156
*Information measure of correlation*		-0.869		-0.827	<0.001*
*Inverse difference normalized*		0.9994		0.9995	<0. 01*
*Inverse difference moment*		0.99980		0.99987	<0.001*

* *p*-value<0.05 indicates a statistically significant difference.

**Table 3:** The performance comparisons of intensity features, texture features, and the combination of both feature sets.

	Intensity	Texture	Combined	Combined vs. Intensity (*p*-value)	Combined vs. Texture (*p*-value)
Accuracy	91% (175/191)	89% (171/191)	92% (176/191)	0.8514	0.3752
Sensitivity	92% (94/102)	89% (91/102)	91% (93/102)	0.8000	0.6377
Specificity	91% (81/89)	89% (80/89)	93% (83/89)	0.5776	0.4183
PPV	92% (94/102)	91% (91/100)	93% (93/99)	0.6197	0.4323
NPV	91% (81/89)	87% (80/91)	90% (83/92)	0.8548	0.6172
Az	0.9682	0.9469	0.9694	0.9701	0.0610

**Fig 3:**
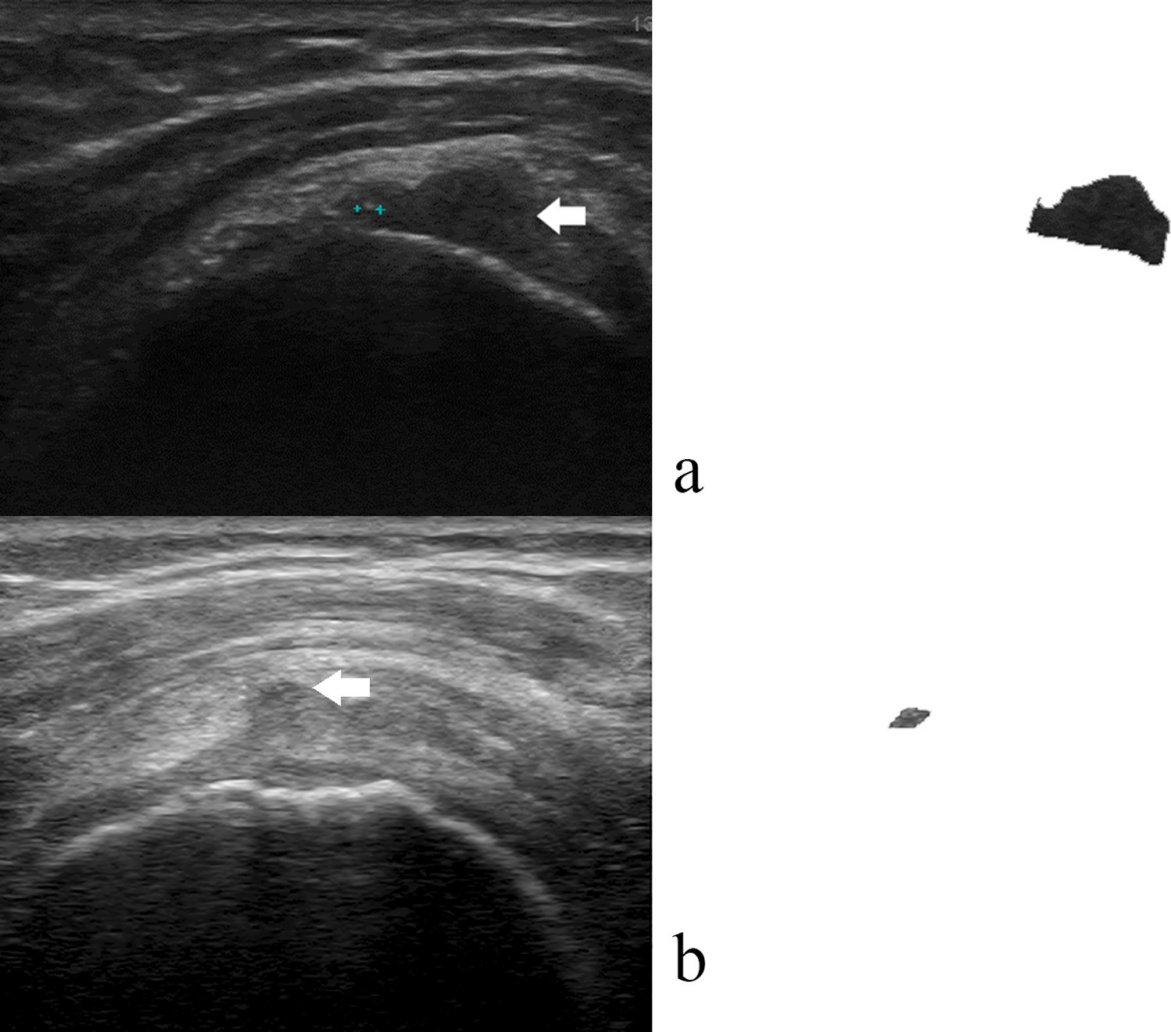
Tear classification results with probabilities higher than 50% were classified to be tear. (a) a moderate supraspinatus near full thickness tear with unobvious characteristics in the ultrasound image (hypoechoic area near the tendon insertion indicated by a white arrow) was misclassified by the texture feature set (42%) but correctly classified by the combination of texture and intensity feature sets (100%). (b) a small supraspinatus partial thickness tear at bursal surface was misclassified by the intensity feature set (9%) but correctly classified by the combination of texture and intensity feature sets (68%).

**Fig 4:**
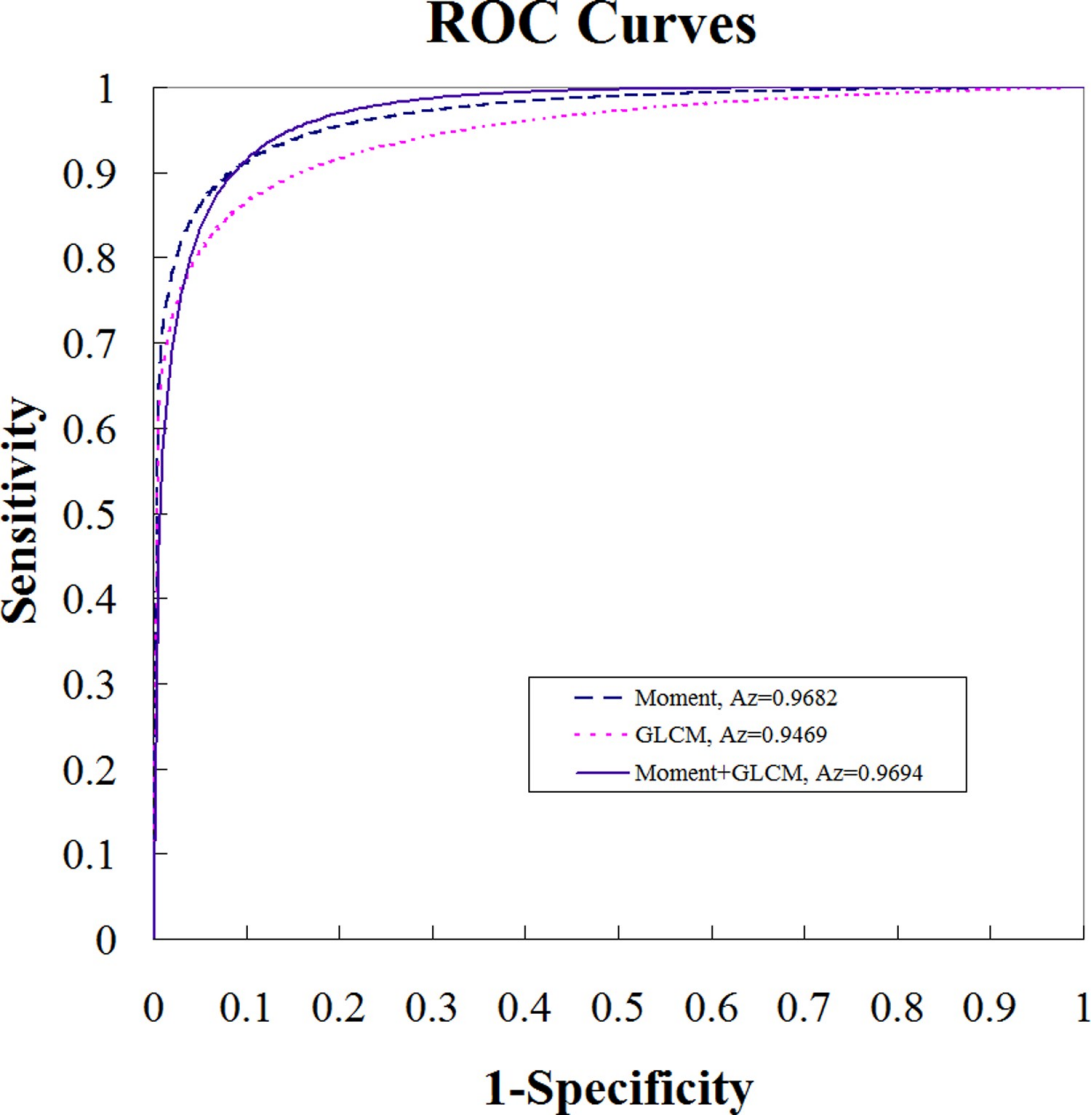
The trade-offs between the sensitivity and specificity of the computer-aided tear classification system using different feature sets are illustrated by receiver operating characteristic curves. (“Moment” is referred to intensity model; “GLCM” is referred to texture model; and “Moment + GLCM” is referred to a combined model.)

Fig 3 demonstrates a supraspinatus tear case that was misclassified by the texture feature set but correctly classified by the combination of texture and intensity feature sets. In Fig 4, the trade-offs between sensitivity and specificity are illustrated using ROC curves, with corresponding Az values.

## Discussion

The proposed CTC system based on intensity and texture features was established to interpret tissue echogenicities of shoulder ultrasound images. The prediction model built by a logistic regression classifier achieved an accuracy of 92% for identifying rotator cuff tears and tendinopathies. The high accuracy suggests that the proposed CTC system is useful for assessing the presence of rotator cuff tears. The classification result was obtained via leave-one-out cross-validation due to the limited cases. The accuracy presented in this study provides us a direction that the proposed CTC system works well in tear classification while the morphology features are useless for differentiation in the observation. With respect to the selected features, Tears tend to be darker due to its higher value of mean intensity and centralized with higher kurtosis value. Besides, high *Inverse difference normalized*, and *Inverse difference moment* mean the gray-scale distribution is uniform and lacking variance.

The proposed CTC system focused on the diagnosis of supraspinatus tears. It is believed that ultrasound has a high diagnostic accuracy for calcific tendinitis, although few studies have assessed this [16, 38]. Calcific tendinitis has several forms, including microcalcification, large soft calcification without acoustic shadow, and large hard calcification appearing as hyperechoic convex with acoustic shadows. All forms of calcific tendinitis of supraspinatus are detected with little difficulty through ultrasound examination [32]. A limitation of the clinical practice of shoulder ultrasound is the inter-observer variability in diagnosing rotator cuff tears. The inter-observer agreement of diagnosing rotator cuff tears, especially partial thickness tears, is only poor to moderate [10] and should be improved for clinical application of the shoulder ultrasound. Therefore, this study proposed a customized CTC system for the diagnosis of rotator cuff tears. Although how much improvement of inter-operator variability was not presented in this study. Future experiment will be performed with scheduled people and time. A previous study used a portion of the lesion (30×60 pixels) for tissue characterization to classify 80 rotator cuff lesions into groups and achieved 92.5% accuracy [39]. The numerous features used in the experiment included the fractal dimension, the texture spectrum, the statistical feature matrix, the texture feature coding method, and the gray-level co-occurrence matrix. Compared to the previous study, our system collected the whole lesion area of 102 tears rather than a sub-region of 20 tears to provide more representative distribution for the evaluation. Using only intensity and texture features in this study is expected to be more efficiency for clinical use. With the proposed system, promising recommendations can be presented to different operator professionals with varying experiences in identifying rotator cuff tears and tendinopathy. The diagnosis of rotator cuff full-thickness tears influences the decision to undertake the surgical repair or arthroplasty.

In the literature, the assessment of full thickness rotator cuff tears is shown to have better sensitivity and specificity compared to partial-thickness rotator cuff tears [20, 40, 41]. A controversial and uncertain issue is whether the accuracy of ultrasound examinations for the assessment of partial-thickness rotator cuff tears is sufficient [16, 42, 43]. With ultrasound examinations, partial-thickness tears are diagnosed when there is a focal hypoechoic or anechoic defect inside of the tendon, involving either the articular or the bursal surface and manifested in at least two perpendicular planes. Limited knowledge of anatomy, inexperience with examination techniques, and patient-related factors (e.g., obesity or muscularity and limited shoulder motion) limit the diagnostic accuracy of partial-thickness rotator cuff tears [44]. The accuracy variation of partial thickness rotator cuff based on shoulder ultrasound images limits the current application of shoulder ultrasounds. In the proposed CTC system, whole lesion areas of either partial-thickness or full thickness rotator cuff tears were delineated to extract lesion features. Lesion features of rotator cuff tears, especially for partial-thickness rupture, are thought to provide less reliable diagnostic information with less-experienced physicians. This is also a limitation of the proposed CTC system. In the future experiment, we will explore the possibility of automatically extracting the lesion area for more detailed classification. The current CTC system was proposed to provide consistent and objective recommendations to less-experienced operators for clinical examinations. Lesion areas were delineated by the orthopedic shoulder surgeon and confirmed by the PM&R physician to obtain the consensus to ensure whole lesion area. Further experiments will be necessary to analyze the diagnostic performance based on lesion area delineated from less-experienced operators and even by automatic segmentation to verify the clinical usefulness of the proposed CTC system. Another limitation is that the collected ultrasound images were generated using a consistent setting. The classification result based on the intensity features extracted from these images achieved an accuracy of 91%. According to the result, using intensity features under consistent settings would be useful in tear classification while the system is customized for a specified ultrasound scanner or setting. In other situations, if the system targets at multi-center hospitals with various scanners, a calibration procedure or more intensity-invariant features should be adopted [24].

MR arthrography is considered as the most sensitive and specific technique for diagnosis according to the meta-analysis study when compared to ultrasound and MRI, and MRI and ultrasound are comparable in accuracy [45]. Nevertheless, the indication and convenience of MR arthrography is more limited than ultrasound and is an invasive procedure. Furthermore, not all supraspinatus tears such as partial thickness tears should be treated with surgical or arthroscopic surgery. Sampling error exists if surgical or arthroscopic findings were used as the gold standard. According to previous meta-analysis study, diagnostic accuracy of supraspinatus tears is high while performed by musculoskeletal radiologists and shoulder orthopedic surgeons [20], and the accuracy would be higher with the consensus of the experienced operators. For the reasons specified above, the gold standard was established by the consensus of the experienced shoulder orthopedic surgeon and PM&R physician who specialize musculoskeletal ultrasound in this study.

This study proposed a CTC system which achieved a high accuracy (92%) in identifying rotator cuff tears, including partial and full thickness tears, by analyzing tissue enclosed in the lesion area. The proposed CTC system performed similar performance to the experienced operators in terms of accuracy. According to a meta-analysis of diagnostic accuracy of ultrasound for rotator cuff tears [20], diagnostic accuracy may be greatest when operated by musculoskeletal radiologists, followed by orthopedic surgeons. The pooled sensitivity and specificity under the direction of musculoskeletal radiologists are both 95%. However, sensitivity is lowered to 46% when the diagnosis of partial thickness tears is given by general radiologists or radiographers [46, 47]. Consequently, the proposed CTC system can provide clinical assistance for general radiologists or ultrasonographers who may not have comparable rates of diagnostic accuracy as musculoskeletal radiologists or orthopedic shoulder surgeons [20].

Additional experiments are needed to explore the clinical application of the proposed CTC system. In particular, future research should examine how to utilize the CTC system in clinical examinations to improve the performance of the observer. More specific, meaningful sonographic findings may be needed to convince observers.

In conclusion, this CTC system based on intensity and texture features extracted from the lesion area in shoulder ultrasound images achieved comparable accuracy in identifying rotator cuff tears to musculoskeletal radiologists and orthopedic shoulder surgeons. The diagnostic suggestions generated by the proposed CTC would be practical and promising in clinical assessments.

## Supporting information

S1 Data The delineated regions of rotator cuff tears. (ZIP)
