# Supplementary figures and images for: Quantitative diagnosis of rotator cuff tears based on sonographic pattern recognition (part 2 of 2)
Source: PLoS One. 2019 Feb 28;14(2):e0212741. doi: 10.1371/journal.pone.0212741 (PMC6394937; doi:10.1371/journal.pone.0212741)

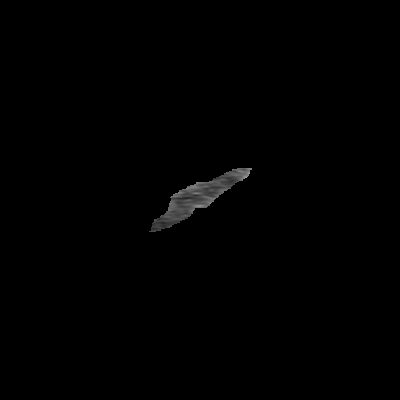

Supplement: S1 Data — (ZIP) [file pone.0212741.s001.zip › Tear/17.bmp]

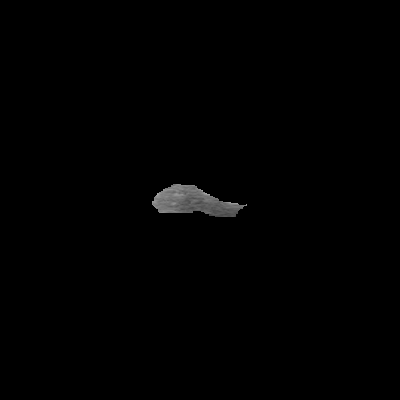

Supplement: S1 Data — (ZIP) [file pone.0212741.s001.zip › Tear/18.bmp]

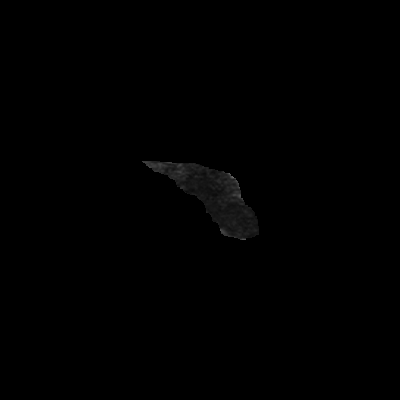

Supplement: S1 Data — (ZIP) [file pone.0212741.s001.zip › Tear/19.bmp]

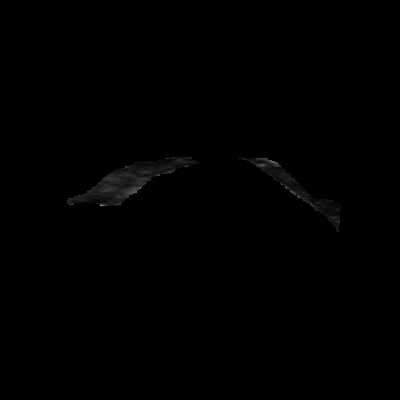

Supplement: S1 Data — (ZIP) [file pone.0212741.s001.zip › Tear/2.bmp]

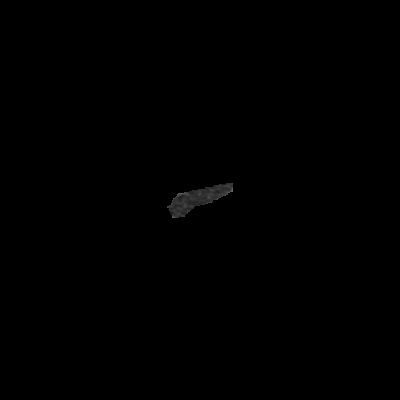

Supplement: S1 Data — (ZIP) [file pone.0212741.s001.zip › Tear/20.bmp]

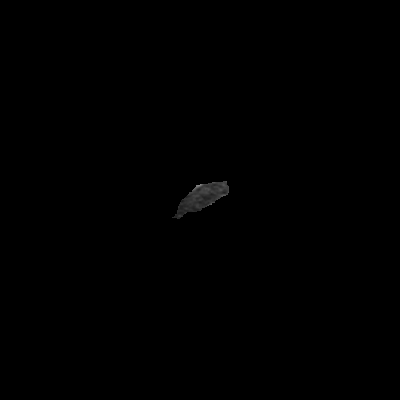

Supplement: S1 Data — (ZIP) [file pone.0212741.s001.zip › Tear/21.bmp]

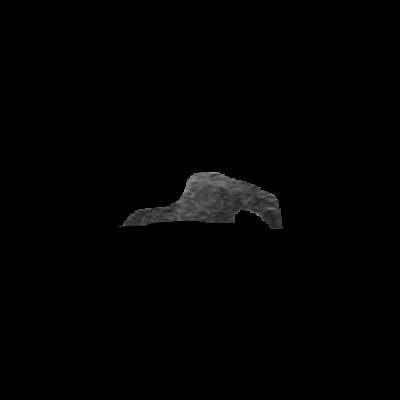

Supplement: S1 Data — (ZIP) [file pone.0212741.s001.zip › Tear/22.bmp]

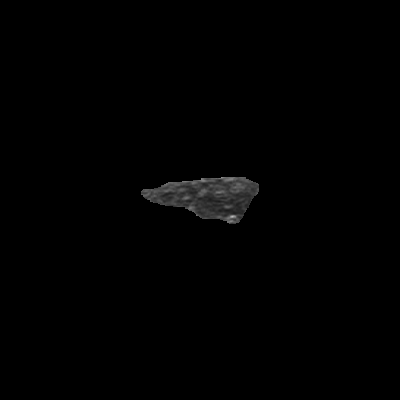

Supplement: S1 Data — (ZIP) [file pone.0212741.s001.zip › Tear/23.bmp]

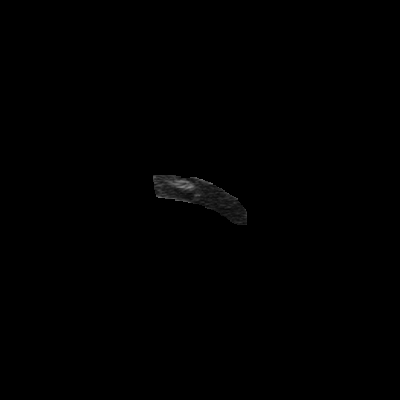

Supplement: S1 Data — (ZIP) [file pone.0212741.s001.zip › Tear/24.bmp]

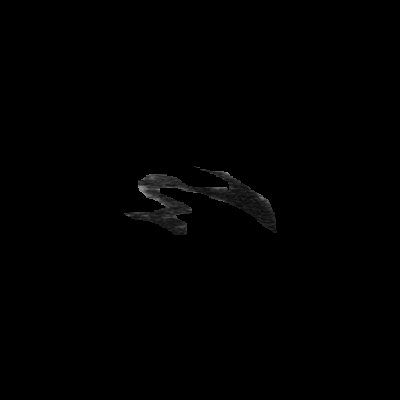

Supplement: S1 Data — (ZIP) [file pone.0212741.s001.zip › Tear/25.bmp]

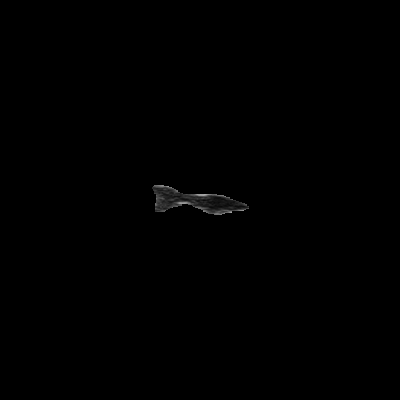

Supplement: S1 Data — (ZIP) [file pone.0212741.s001.zip › Tear/26.bmp]

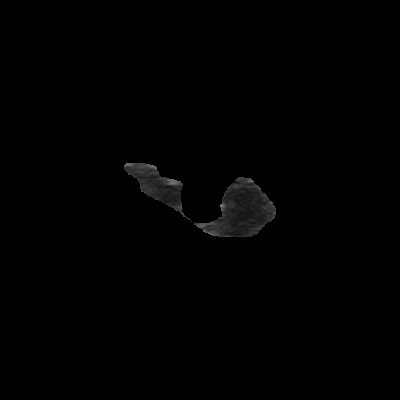

Supplement: S1 Data — (ZIP) [file pone.0212741.s001.zip › Tear/27.bmp]

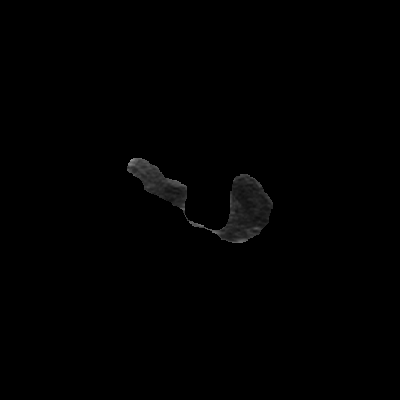

Supplement: S1 Data — (ZIP) [file pone.0212741.s001.zip › Tear/28.bmp]

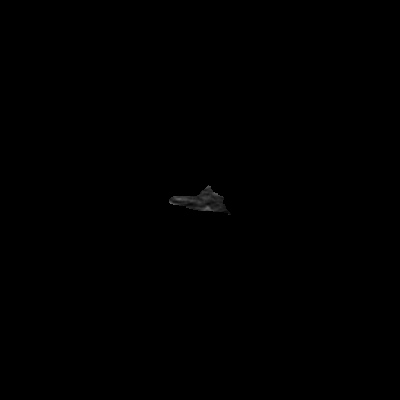

Supplement: S1 Data — (ZIP) [file pone.0212741.s001.zip › Tear/29.bmp]

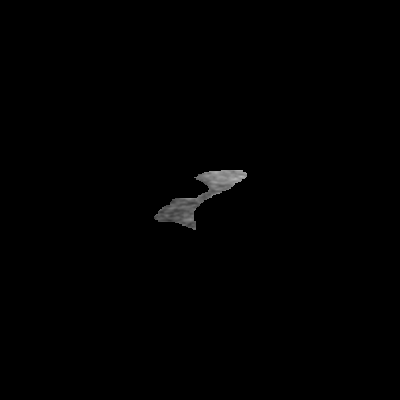

Supplement: S1 Data — (ZIP) [file pone.0212741.s001.zip › Tear/3.bmp]

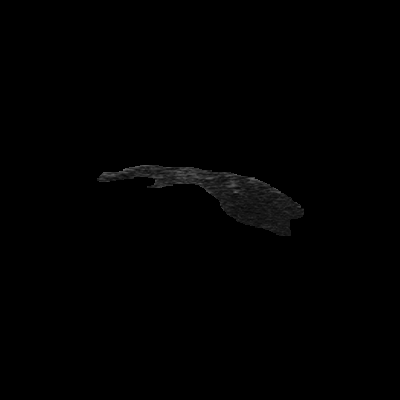

Supplement: S1 Data — (ZIP) [file pone.0212741.s001.zip › Tear/30.bmp]

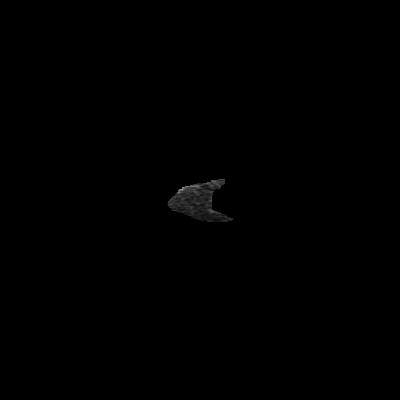

Supplement: S1 Data — (ZIP) [file pone.0212741.s001.zip › Tear/31.bmp]

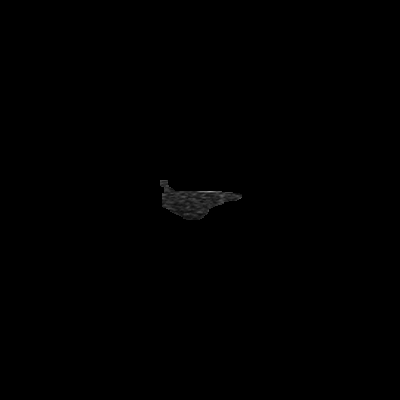

Supplement: S1 Data — (ZIP) [file pone.0212741.s001.zip › Tear/32.bmp]

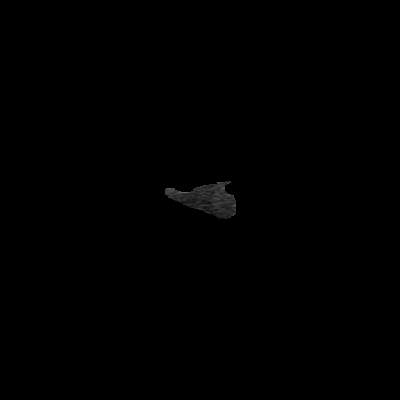

Supplement: S1 Data — (ZIP) [file pone.0212741.s001.zip › Tear/33.bmp]

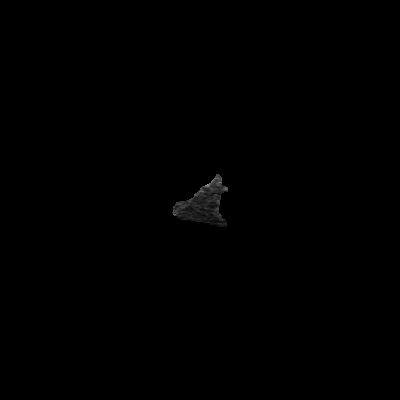

Supplement: S1 Data — (ZIP) [file pone.0212741.s001.zip › Tear/34.bmp]

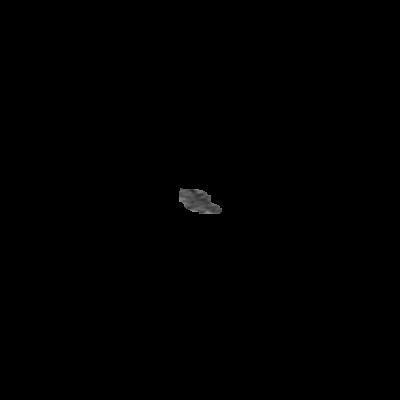

Supplement: S1 Data — (ZIP) [file pone.0212741.s001.zip › Tear/35.bmp]

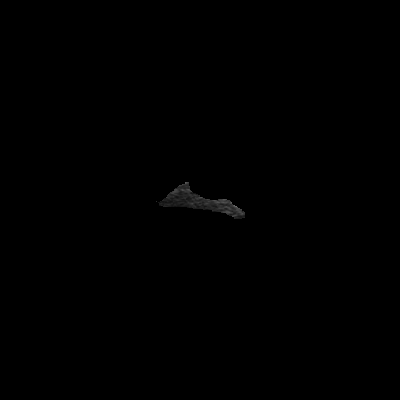

Supplement: S1 Data — (ZIP) [file pone.0212741.s001.zip › Tear/36.bmp]

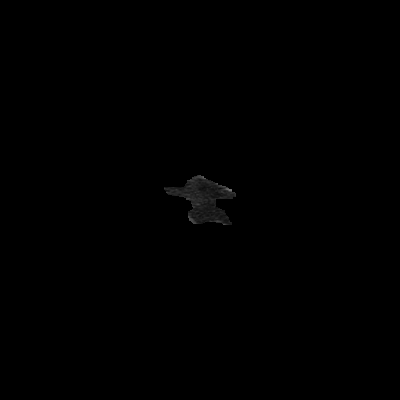

Supplement: S1 Data — (ZIP) [file pone.0212741.s001.zip › Tear/37.bmp]

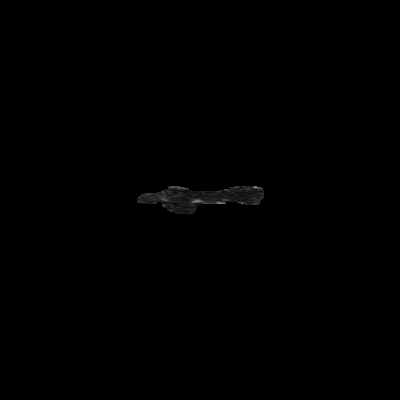

Supplement: S1 Data — (ZIP) [file pone.0212741.s001.zip › Tear/38.bmp]

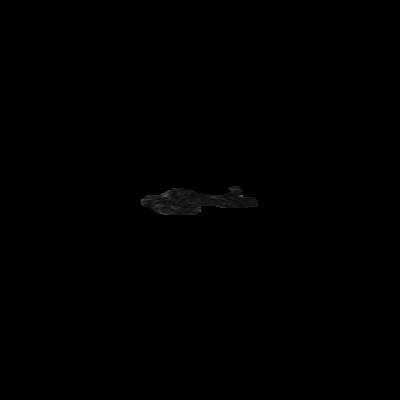

Supplement: S1 Data — (ZIP) [file pone.0212741.s001.zip › Tear/39.bmp]

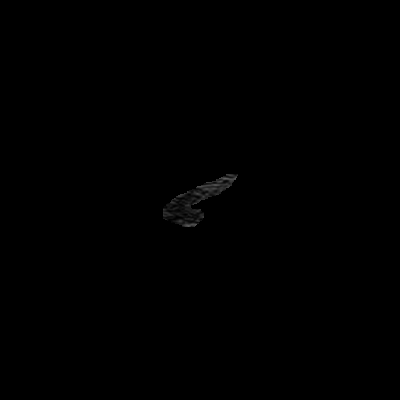

Supplement: S1 Data — (ZIP) [file pone.0212741.s001.zip › Tear/4.bmp]

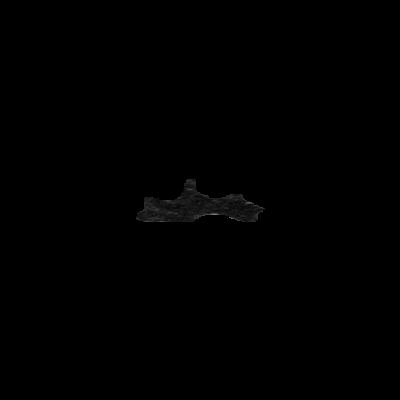

Supplement: S1 Data — (ZIP) [file pone.0212741.s001.zip › Tear/40.bmp]

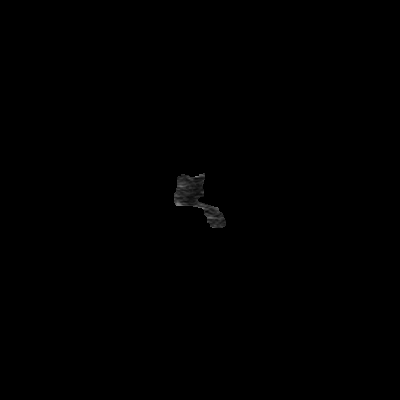

Supplement: S1 Data — (ZIP) [file pone.0212741.s001.zip › Tear/41.bmp]

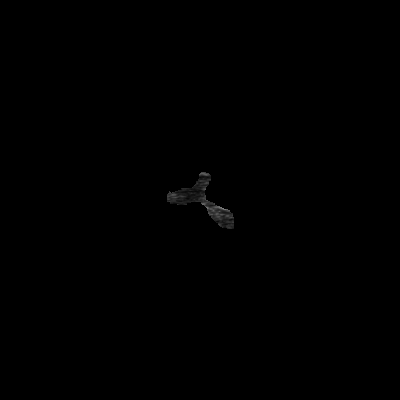

Supplement: S1 Data — (ZIP) [file pone.0212741.s001.zip › Tear/42.bmp]

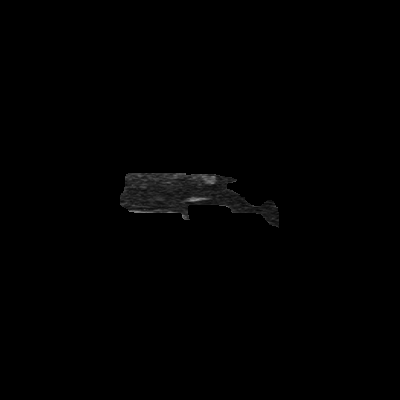

Supplement: S1 Data — (ZIP) [file pone.0212741.s001.zip › Tear/43.bmp]

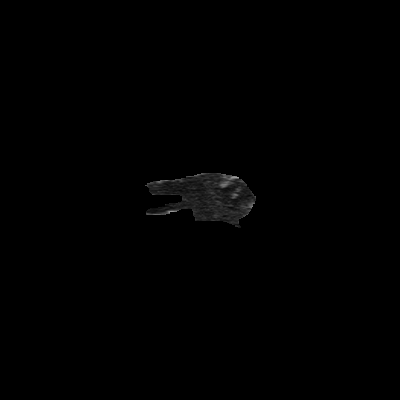

Supplement: S1 Data — (ZIP) [file pone.0212741.s001.zip › Tear/44.bmp]

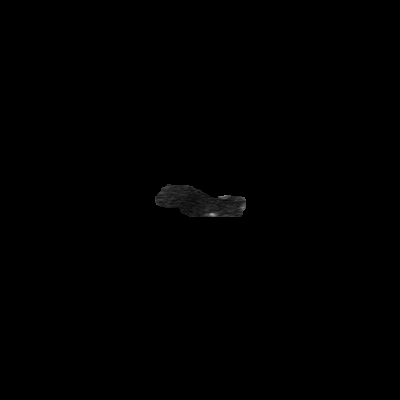

Supplement: S1 Data — (ZIP) [file pone.0212741.s001.zip › Tear/45.bmp]

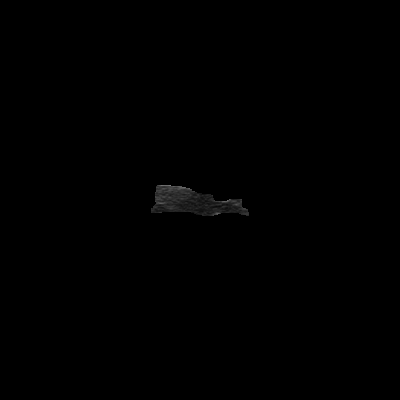

Supplement: S1 Data — (ZIP) [file pone.0212741.s001.zip › Tear/46.bmp]

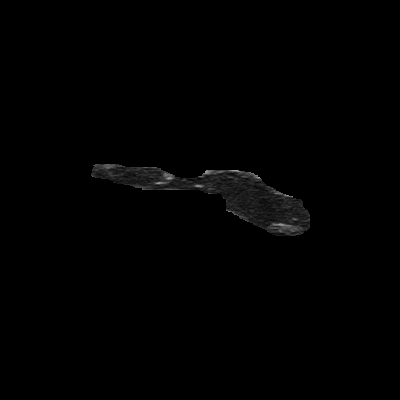

Supplement: S1 Data — (ZIP) [file pone.0212741.s001.zip › Tear/47.bmp]

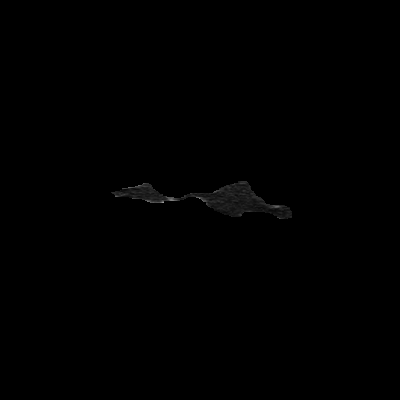

Supplement: S1 Data — (ZIP) [file pone.0212741.s001.zip › Tear/48.bmp]

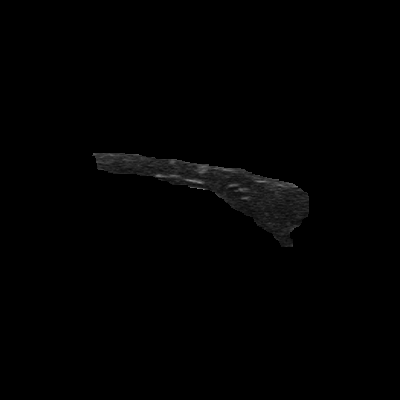

Supplement: S1 Data — (ZIP) [file pone.0212741.s001.zip › Tear/49.bmp]

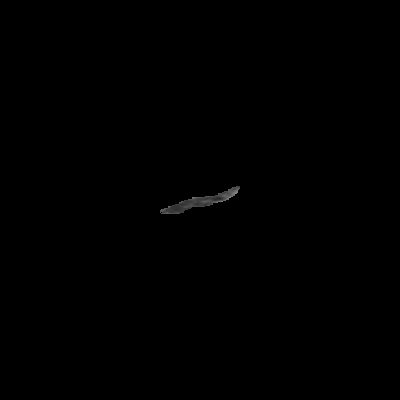

Supplement: S1 Data — (ZIP) [file pone.0212741.s001.zip › Tear/5.bmp]

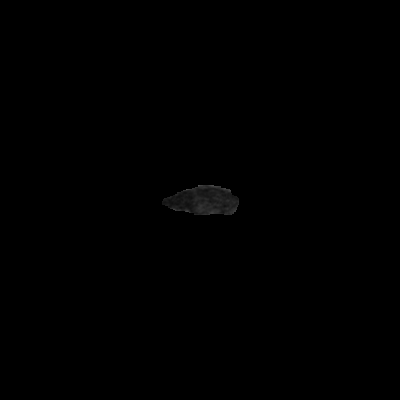

Supplement: S1 Data — (ZIP) [file pone.0212741.s001.zip › Tear/50.bmp]

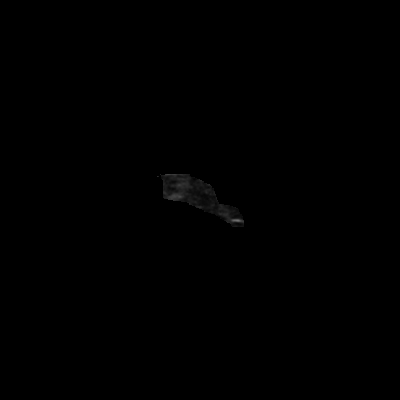

Supplement: S1 Data — (ZIP) [file pone.0212741.s001.zip › Tear/51.bmp]

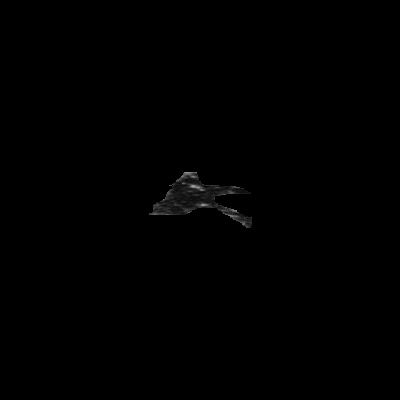

Supplement: S1 Data — (ZIP) [file pone.0212741.s001.zip › Tear/52.bmp]

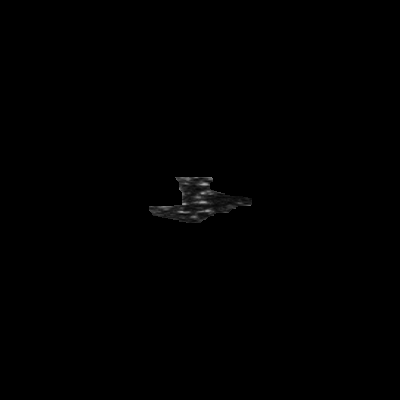

Supplement: S1 Data — (ZIP) [file pone.0212741.s001.zip › Tear/53.bmp]

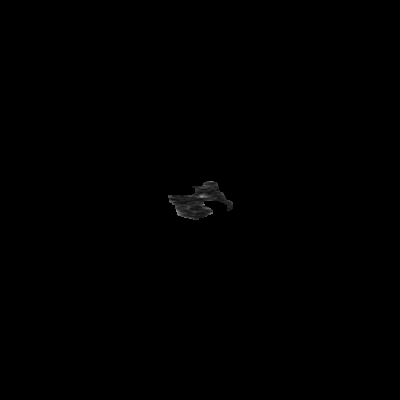

Supplement: S1 Data — (ZIP) [file pone.0212741.s001.zip › Tear/54.bmp]

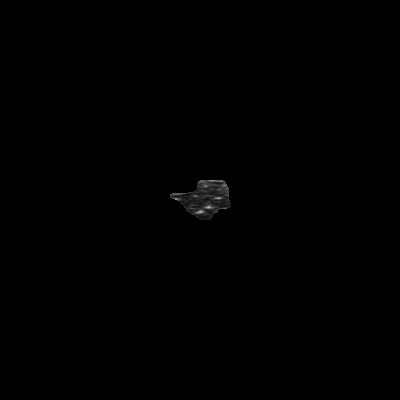

Supplement: S1 Data — (ZIP) [file pone.0212741.s001.zip › Tear/55.bmp]

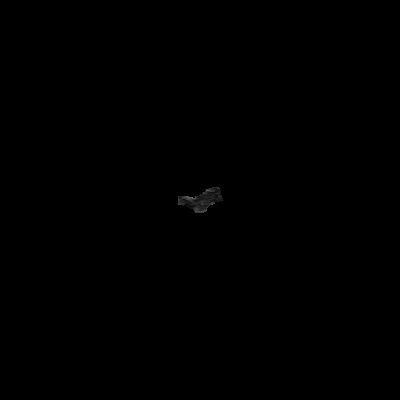

Supplement: S1 Data — (ZIP) [file pone.0212741.s001.zip › Tear/56.bmp]

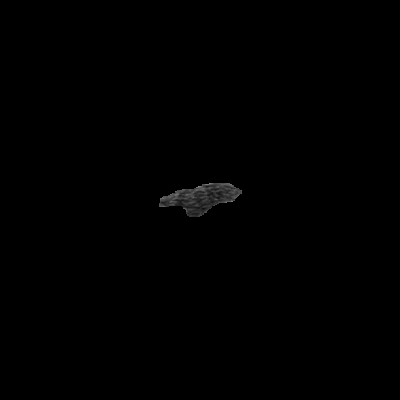

Supplement: S1 Data — (ZIP) [file pone.0212741.s001.zip › Tear/57.bmp]

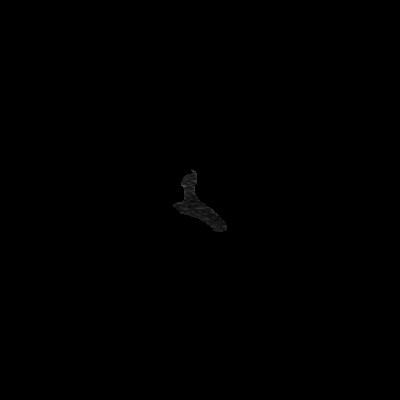

Supplement: S1 Data — (ZIP) [file pone.0212741.s001.zip › Tear/58.bmp]

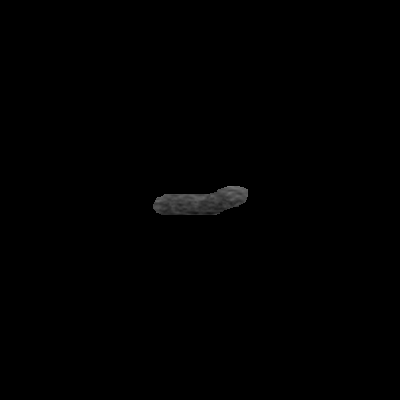

Supplement: S1 Data — (ZIP) [file pone.0212741.s001.zip › Tear/59.bmp]

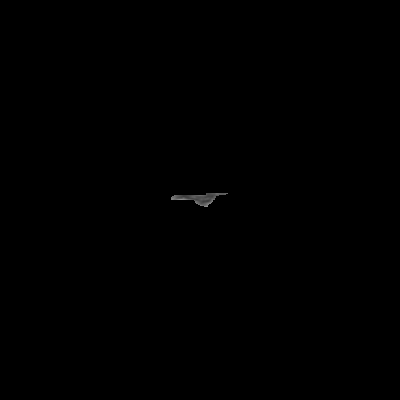

Supplement: S1 Data — (ZIP) [file pone.0212741.s001.zip › Tear/6.bmp]

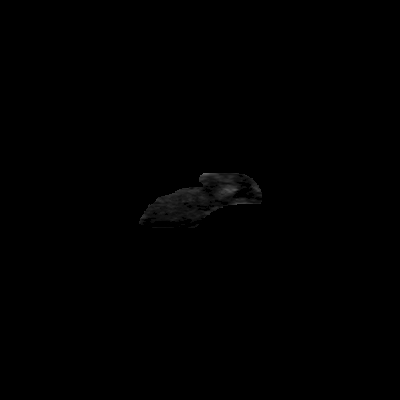

Supplement: S1 Data — (ZIP) [file pone.0212741.s001.zip › Tear/60.bmp]

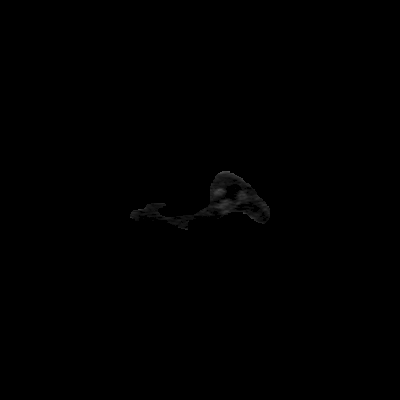

Supplement: S1 Data — (ZIP) [file pone.0212741.s001.zip › Tear/61.bmp]

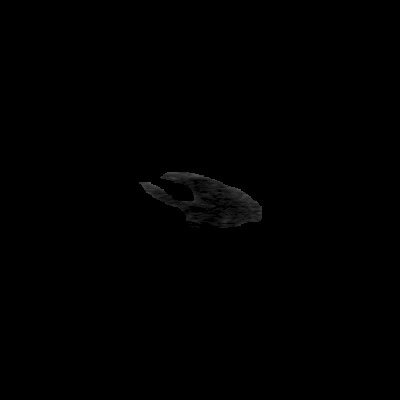

Supplement: S1 Data — (ZIP) [file pone.0212741.s001.zip › Tear/62.bmp]

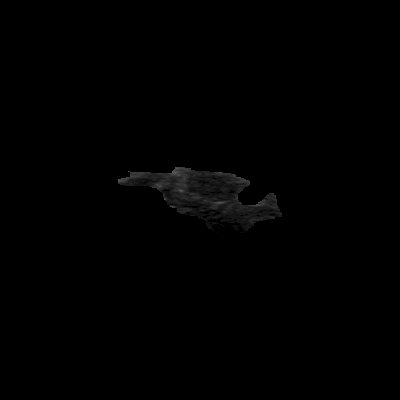

Supplement: S1 Data — (ZIP) [file pone.0212741.s001.zip › Tear/63.bmp]

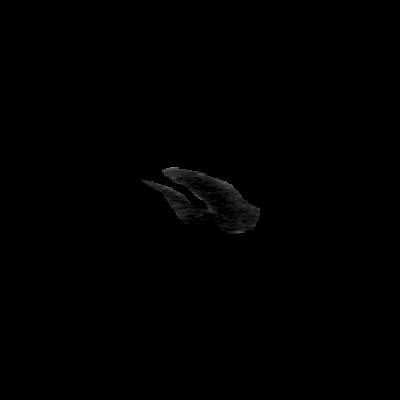

Supplement: S1 Data — (ZIP) [file pone.0212741.s001.zip › Tear/64.bmp]

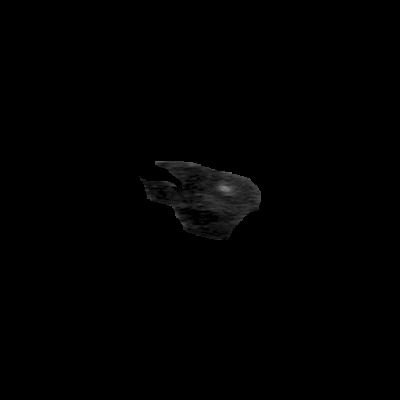

Supplement: S1 Data — (ZIP) [file pone.0212741.s001.zip › Tear/65.bmp]

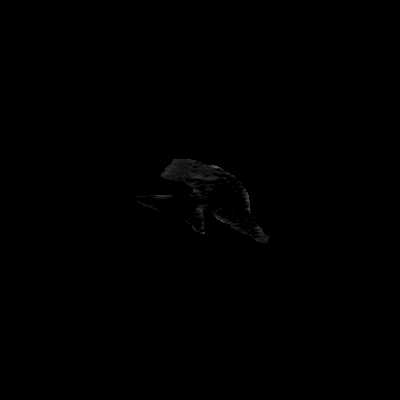

Supplement: S1 Data — (ZIP) [file pone.0212741.s001.zip › Tear/66.bmp]

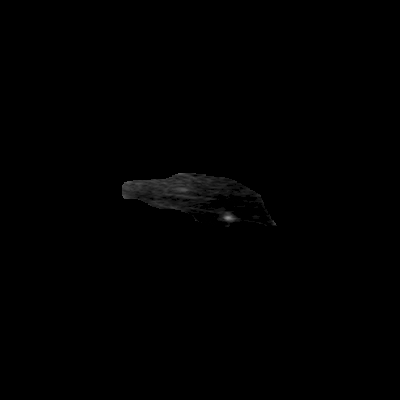

Supplement: S1 Data — (ZIP) [file pone.0212741.s001.zip › Tear/67.bmp]

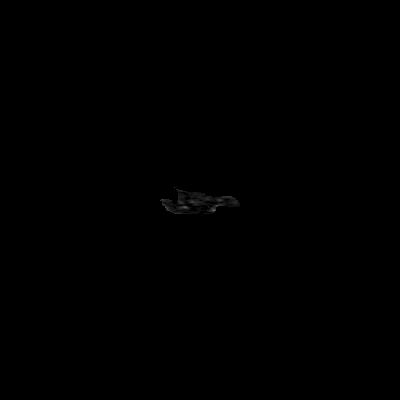

Supplement: S1 Data — (ZIP) [file pone.0212741.s001.zip › Tear/68.bmp]

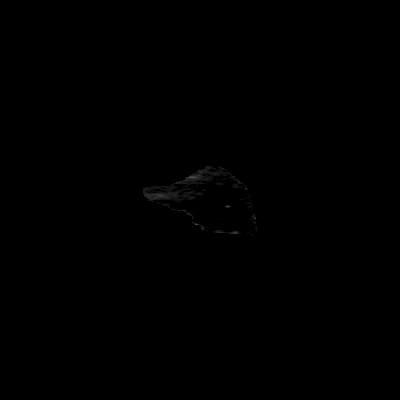

Supplement: S1 Data — (ZIP) [file pone.0212741.s001.zip › Tear/69.bmp]

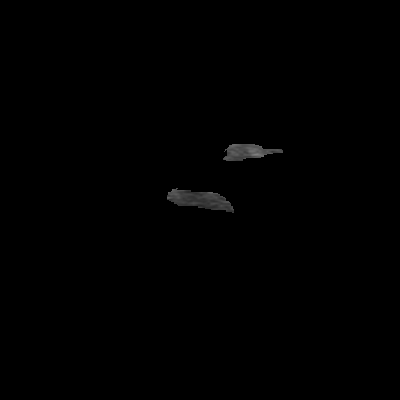

Supplement: S1 Data — (ZIP) [file pone.0212741.s001.zip › Tear/7.bmp]

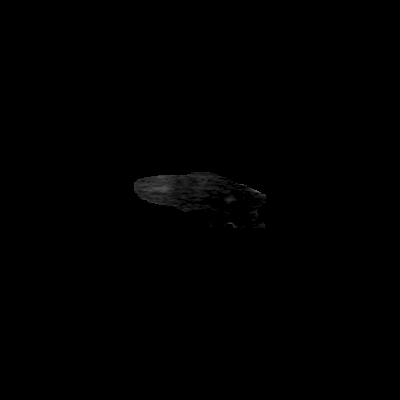

Supplement: S1 Data — (ZIP) [file pone.0212741.s001.zip › Tear/70.bmp]

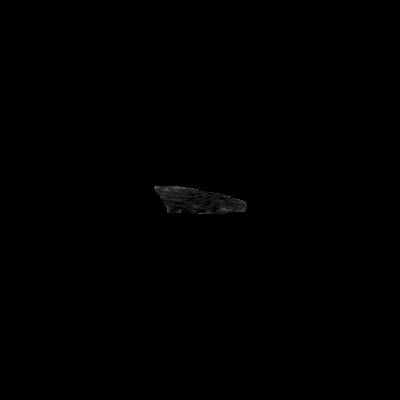

Supplement: S1 Data — (ZIP) [file pone.0212741.s001.zip › Tear/71.bmp]

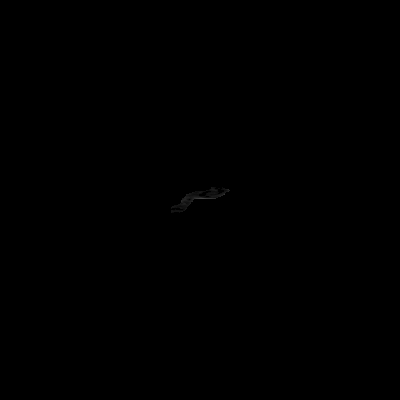

Supplement: S1 Data — (ZIP) [file pone.0212741.s001.zip › Tear/72.bmp]

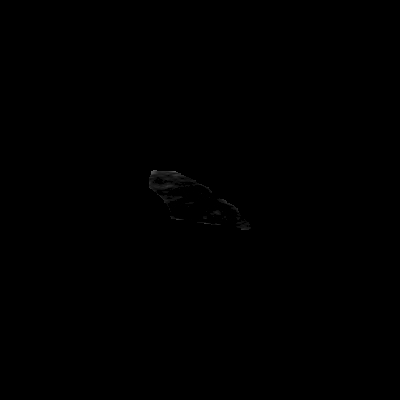

Supplement: S1 Data — (ZIP) [file pone.0212741.s001.zip › Tear/73.bmp]

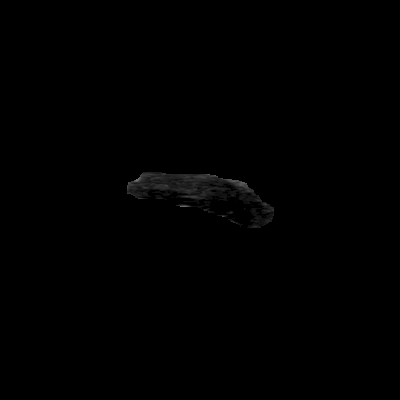

Supplement: S1 Data — (ZIP) [file pone.0212741.s001.zip › Tear/74.bmp]

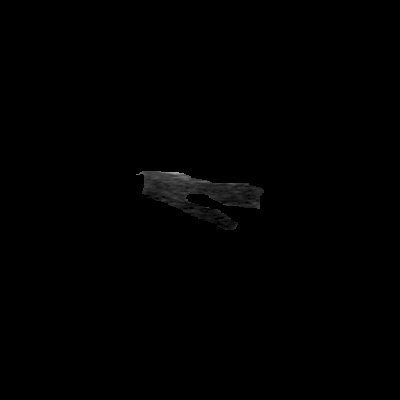

Supplement: S1 Data — (ZIP) [file pone.0212741.s001.zip › Tear/75.bmp]

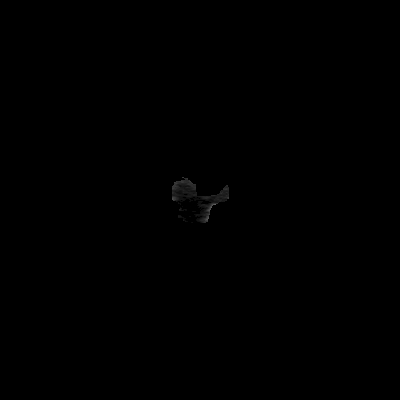

Supplement: S1 Data — (ZIP) [file pone.0212741.s001.zip › Tear/76.bmp]

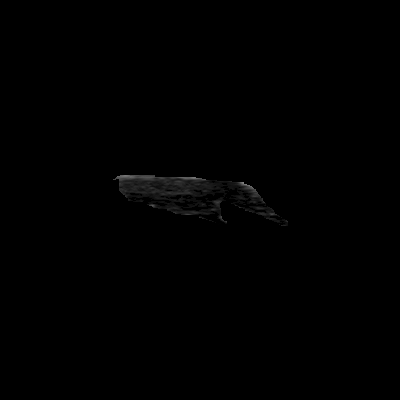

Supplement: S1 Data — (ZIP) [file pone.0212741.s001.zip › Tear/77.bmp]

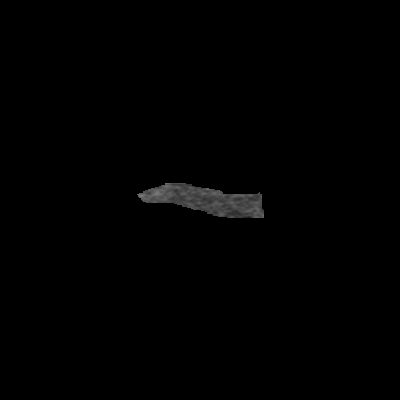

Supplement: S1 Data — (ZIP) [file pone.0212741.s001.zip › Tear/78.bmp]

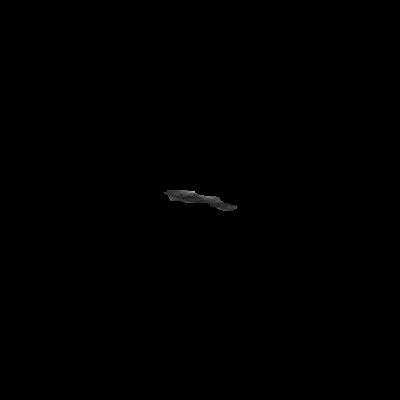

Supplement: S1 Data — (ZIP) [file pone.0212741.s001.zip › Tear/79.bmp]

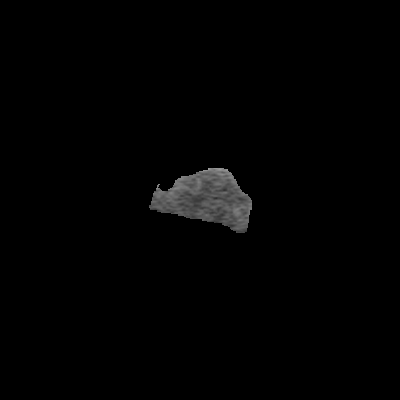

Supplement: S1 Data — (ZIP) [file pone.0212741.s001.zip › Tear/8.bmp]

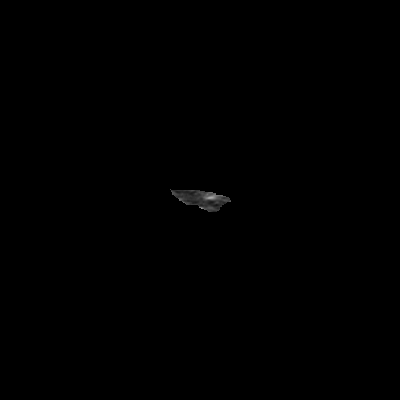

Supplement: S1 Data — (ZIP) [file pone.0212741.s001.zip › Tear/80.bmp]

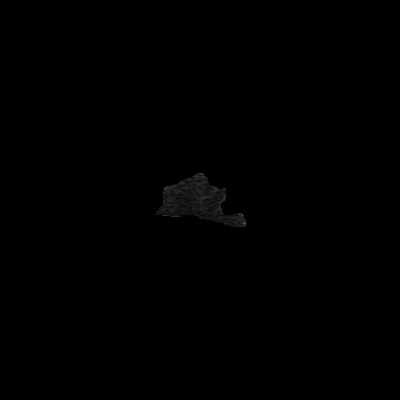

Supplement: S1 Data — (ZIP) [file pone.0212741.s001.zip › Tear/81.bmp]

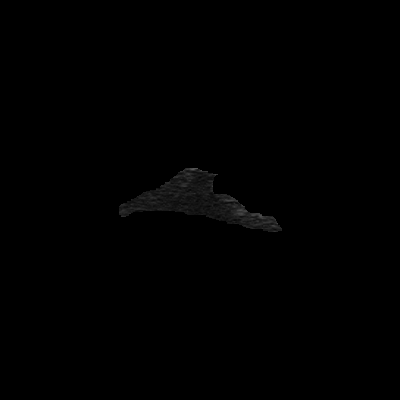

Supplement: S1 Data — (ZIP) [file pone.0212741.s001.zip › Tear/82.bmp]

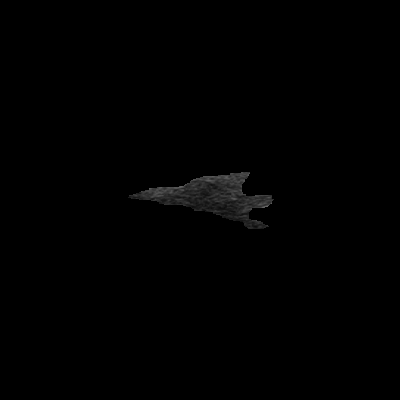

Supplement: S1 Data — (ZIP) [file pone.0212741.s001.zip › Tear/83.bmp]

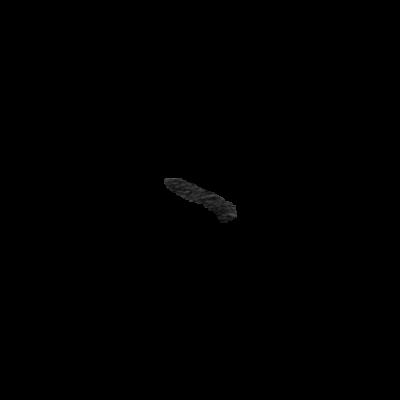

Supplement: S1 Data — (ZIP) [file pone.0212741.s001.zip › Tear/84.bmp]

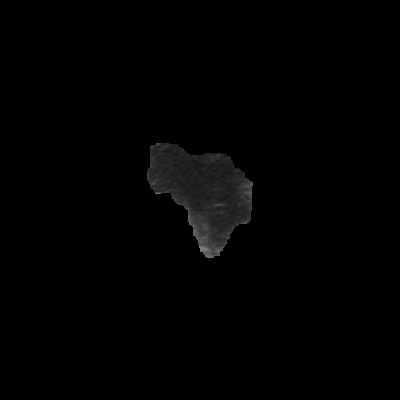

Supplement: S1 Data — (ZIP) [file pone.0212741.s001.zip › Tear/85.bmp]

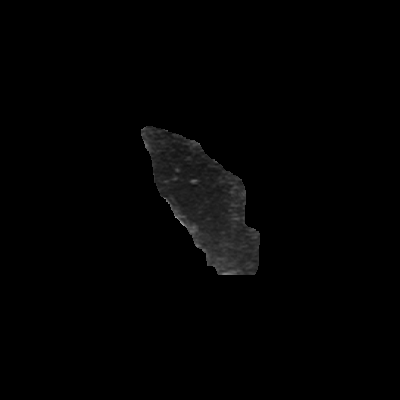

Supplement: S1 Data — (ZIP) [file pone.0212741.s001.zip › Tear/86.bmp]

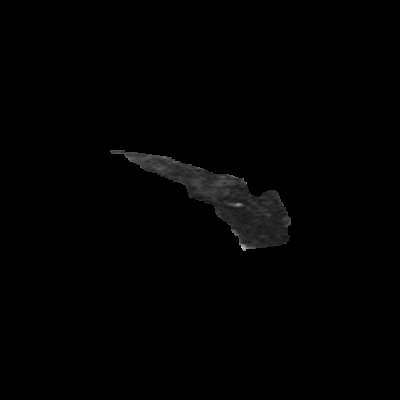

Supplement: S1 Data — (ZIP) [file pone.0212741.s001.zip › Tear/87.bmp]

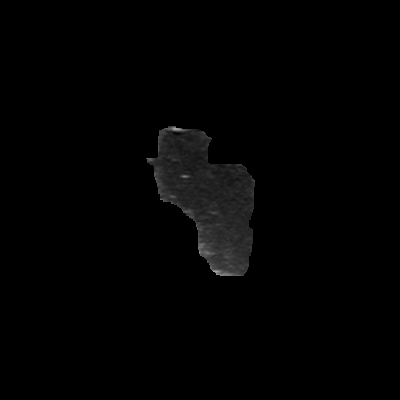

Supplement: S1 Data — (ZIP) [file pone.0212741.s001.zip › Tear/88.bmp]

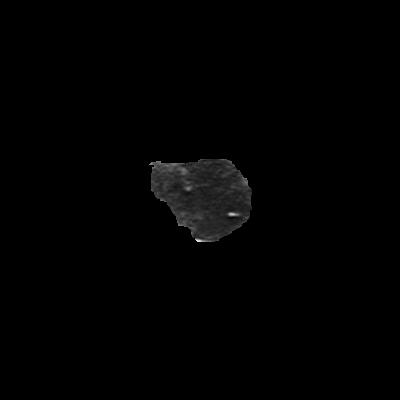

Supplement: S1 Data — (ZIP) [file pone.0212741.s001.zip › Tear/89.bmp]

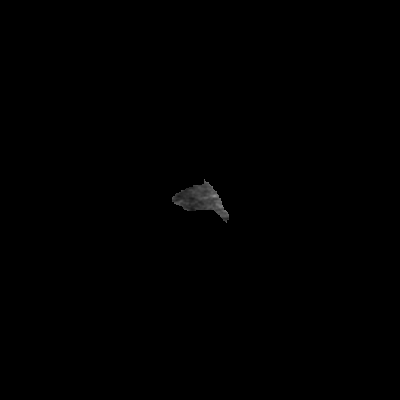

Supplement: S1 Data — (ZIP) [file pone.0212741.s001.zip › Tear/9.bmp]

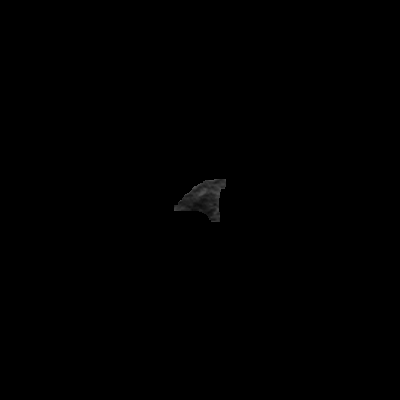

Supplement: S1 Data — (ZIP) [file pone.0212741.s001.zip › Tear/90.bmp]

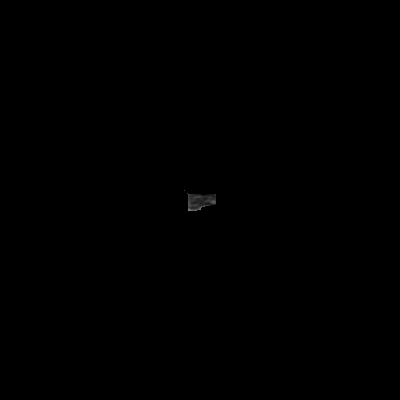

Supplement: S1 Data — (ZIP) [file pone.0212741.s001.zip › Tear/91.bmp]

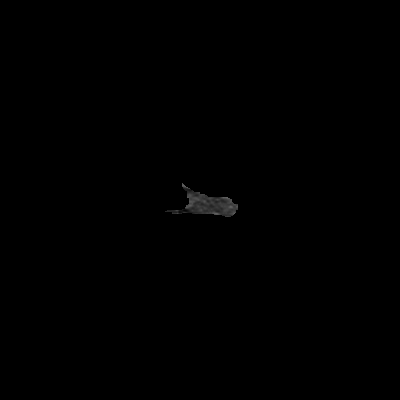

Supplement: S1 Data — (ZIP) [file pone.0212741.s001.zip › Tear/92.bmp]

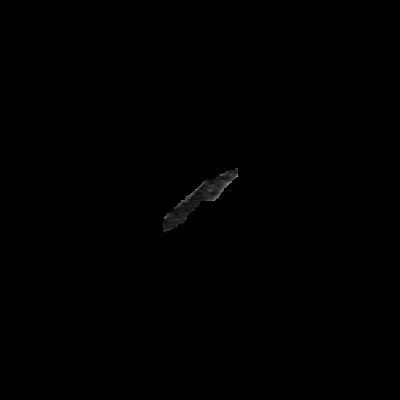

Supplement: S1 Data — (ZIP) [file pone.0212741.s001.zip › Tear/93.bmp]

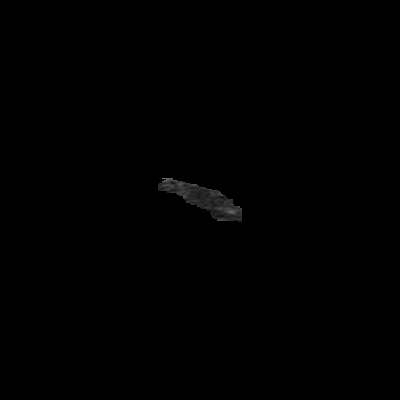

Supplement: S1 Data — (ZIP) [file pone.0212741.s001.zip › Tear/94.bmp]

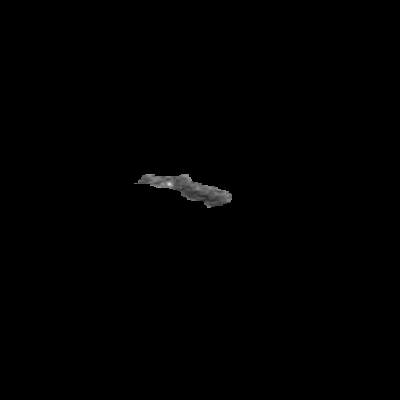

Supplement: S1 Data — (ZIP) [file pone.0212741.s001.zip › Tear/95.bmp]

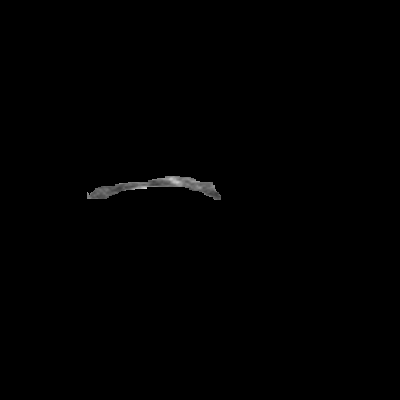

Supplement: S1 Data — (ZIP) [file pone.0212741.s001.zip › Tear/96.bmp]

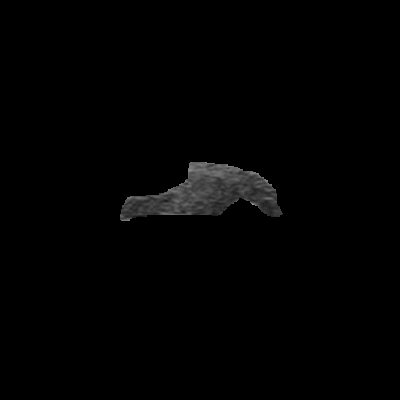

Supplement: S1 Data — (ZIP) [file pone.0212741.s001.zip › Tear/97.bmp]

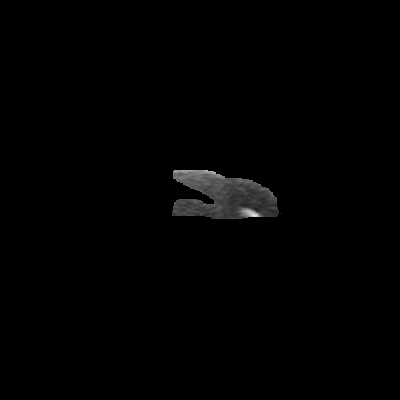

Supplement: S1 Data — (ZIP) [file pone.0212741.s001.zip › Tear/98.bmp]

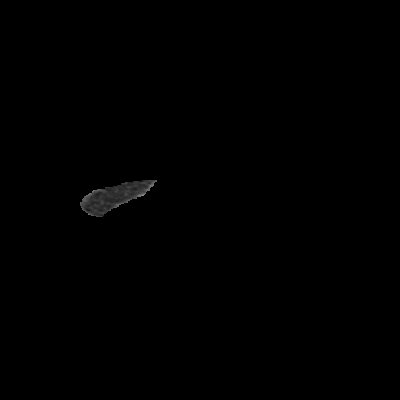

Supplement: S1 Data — (ZIP) [file pone.0212741.s001.zip › Tear/99.bmp]
