# Supplementary figures and images for: Quantitative diagnosis of rotator cuff tears based on sonographic pattern recognition (part 1 of 2)
Source: PLoS One. 2019 Feb 28;14(2):e0212741. doi: 10.1371/journal.pone.0212741 (PMC6394937; doi:10.1371/journal.pone.0212741)

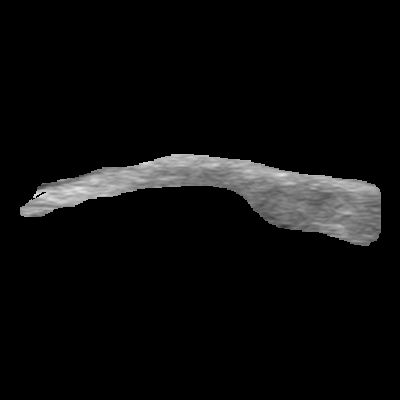

Supplement: S1 Data — (ZIP) [file pone.0212741.s001.zip › NotTear/1.bmp]

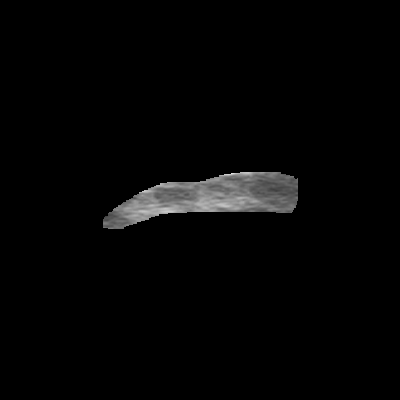

Supplement: S1 Data — (ZIP) [file pone.0212741.s001.zip › NotTear/10.bmp]

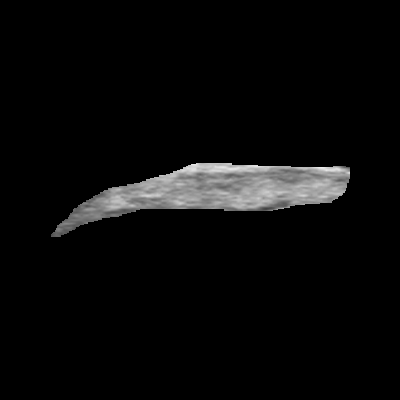

Supplement: S1 Data — (ZIP) [file pone.0212741.s001.zip › NotTear/11.bmp]

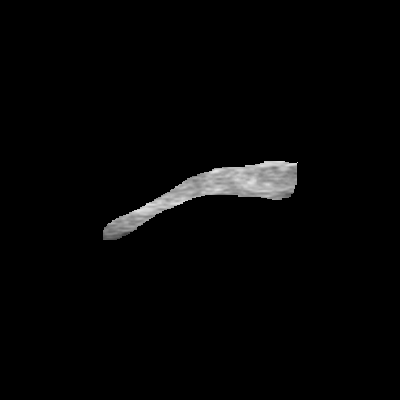

Supplement: S1 Data — (ZIP) [file pone.0212741.s001.zip › NotTear/12.bmp]

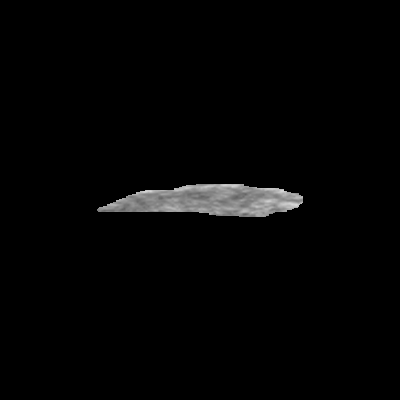

Supplement: S1 Data — (ZIP) [file pone.0212741.s001.zip › NotTear/13.bmp]

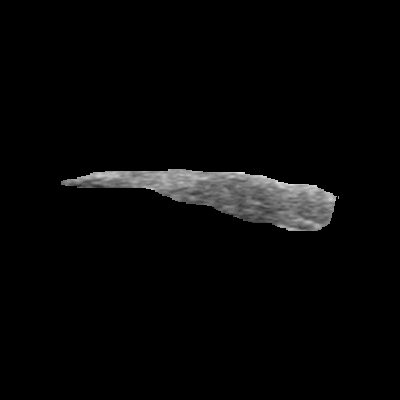

Supplement: S1 Data — (ZIP) [file pone.0212741.s001.zip › NotTear/14.bmp]

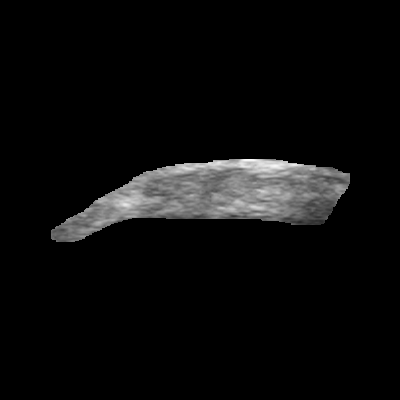

Supplement: S1 Data — (ZIP) [file pone.0212741.s001.zip › NotTear/15.bmp]

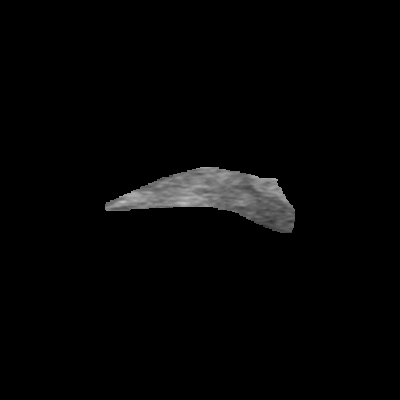

Supplement: S1 Data — (ZIP) [file pone.0212741.s001.zip › NotTear/16.bmp]

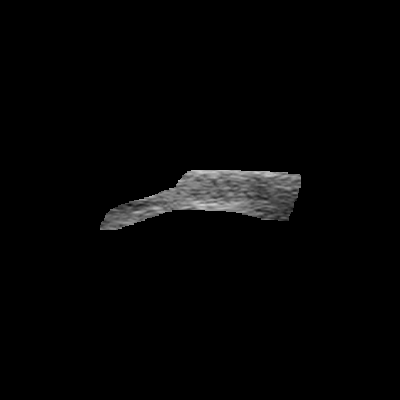

Supplement: S1 Data — (ZIP) [file pone.0212741.s001.zip › NotTear/17.bmp]

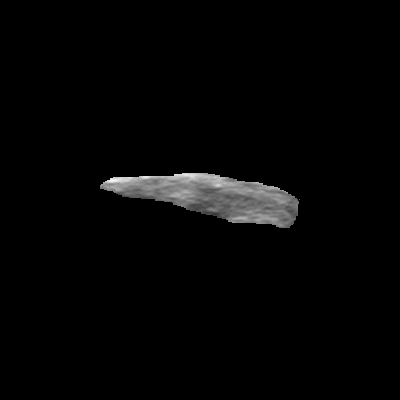

Supplement: S1 Data — (ZIP) [file pone.0212741.s001.zip › NotTear/18.bmp]

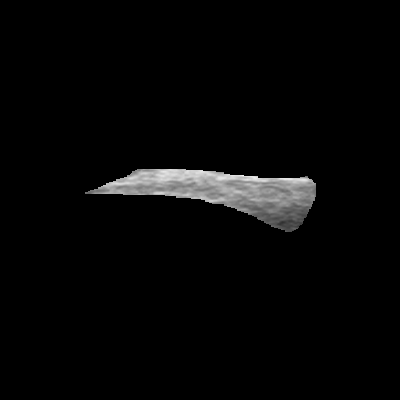

Supplement: S1 Data — (ZIP) [file pone.0212741.s001.zip › NotTear/19.bmp]

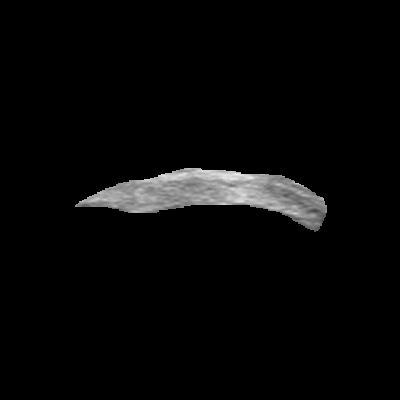

Supplement: S1 Data — (ZIP) [file pone.0212741.s001.zip › NotTear/20.bmp]

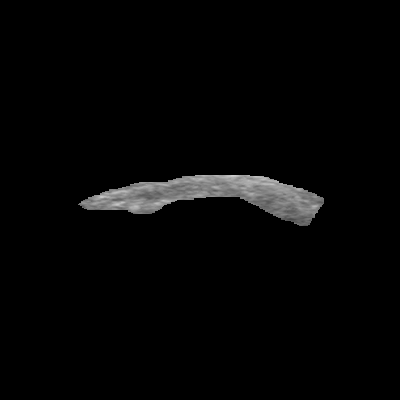

Supplement: S1 Data — (ZIP) [file pone.0212741.s001.zip › NotTear/21.bmp]

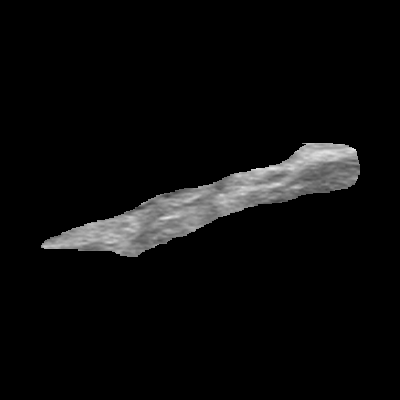

Supplement: S1 Data — (ZIP) [file pone.0212741.s001.zip › NotTear/22.bmp]

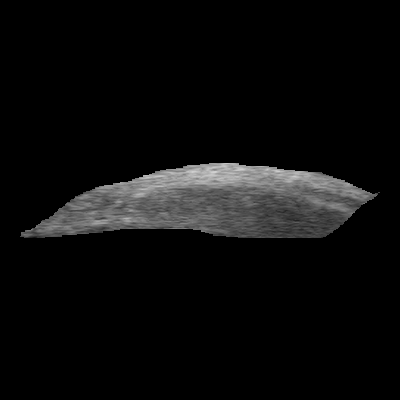

Supplement: S1 Data — (ZIP) [file pone.0212741.s001.zip › NotTear/23.bmp]

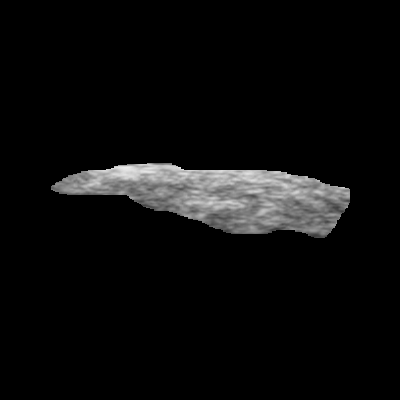

Supplement: S1 Data — (ZIP) [file pone.0212741.s001.zip › NotTear/24.bmp]

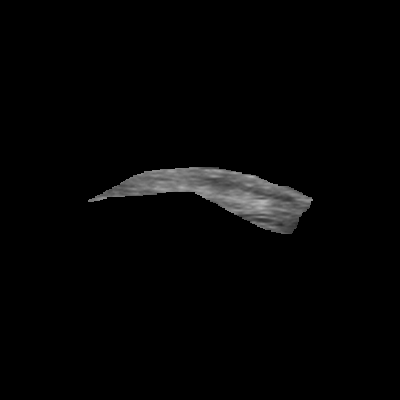

Supplement: S1 Data — (ZIP) [file pone.0212741.s001.zip › NotTear/25.bmp]

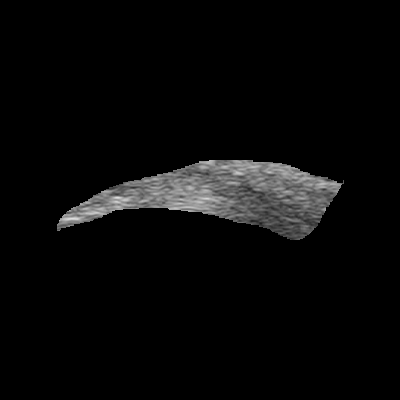

Supplement: S1 Data — (ZIP) [file pone.0212741.s001.zip › NotTear/26.bmp]

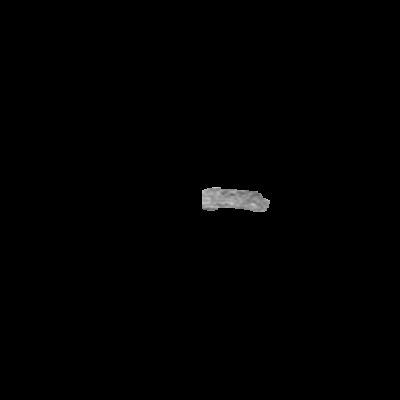

Supplement: S1 Data — (ZIP) [file pone.0212741.s001.zip › NotTear/27.bmp]

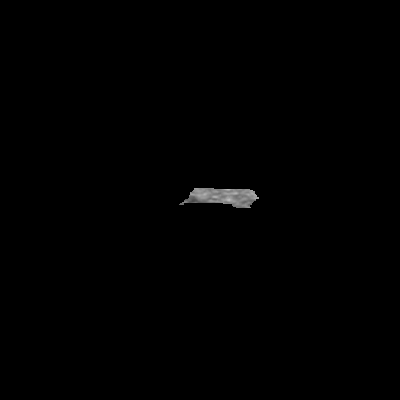

Supplement: S1 Data — (ZIP) [file pone.0212741.s001.zip › NotTear/28.bmp]

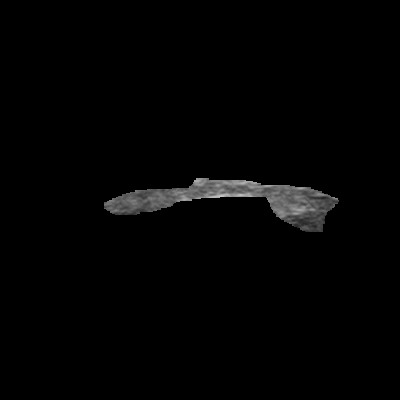

Supplement: S1 Data — (ZIP) [file pone.0212741.s001.zip › NotTear/29.bmp]

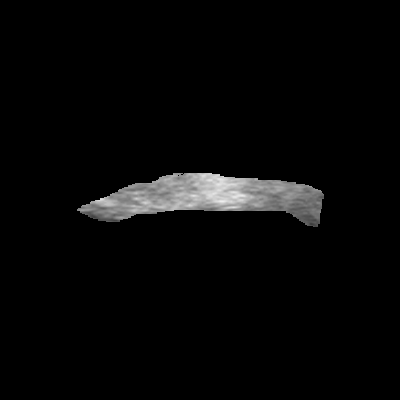

Supplement: S1 Data — (ZIP) [file pone.0212741.s001.zip › NotTear/3.bmp]

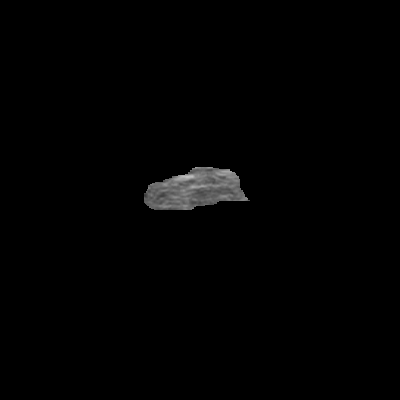

Supplement: S1 Data — (ZIP) [file pone.0212741.s001.zip › NotTear/30.bmp]

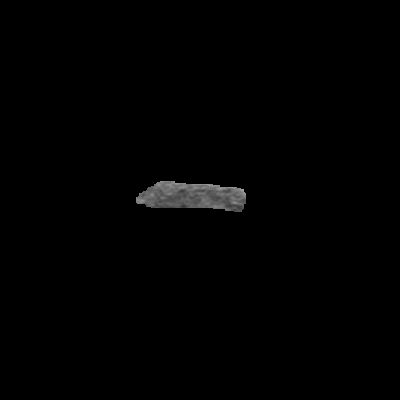

Supplement: S1 Data — (ZIP) [file pone.0212741.s001.zip › NotTear/31.bmp]

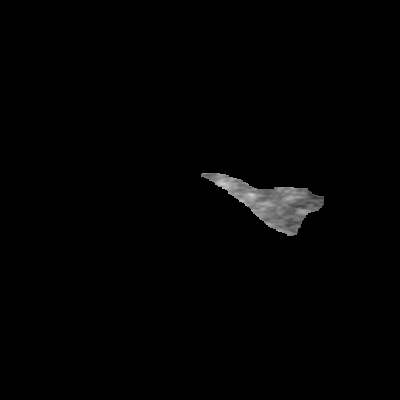

Supplement: S1 Data — (ZIP) [file pone.0212741.s001.zip › NotTear/32.bmp]

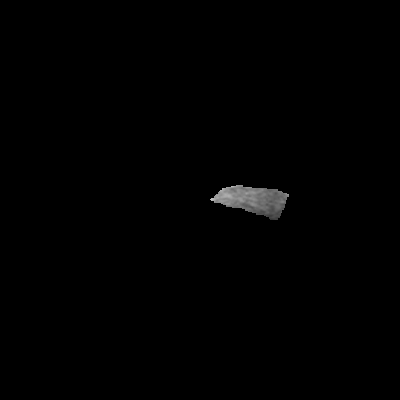

Supplement: S1 Data — (ZIP) [file pone.0212741.s001.zip › NotTear/33.bmp]

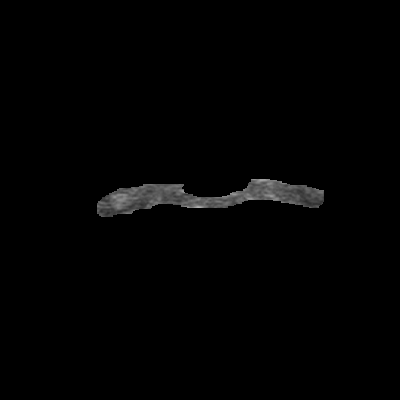

Supplement: S1 Data — (ZIP) [file pone.0212741.s001.zip › NotTear/34.bmp]

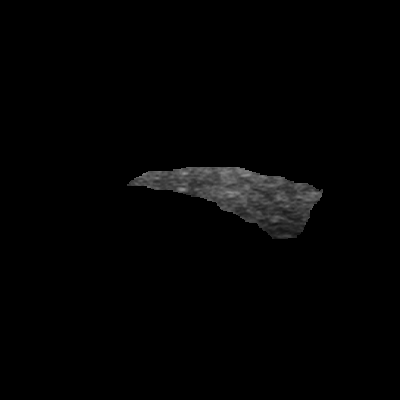

Supplement: S1 Data — (ZIP) [file pone.0212741.s001.zip › NotTear/35.bmp]

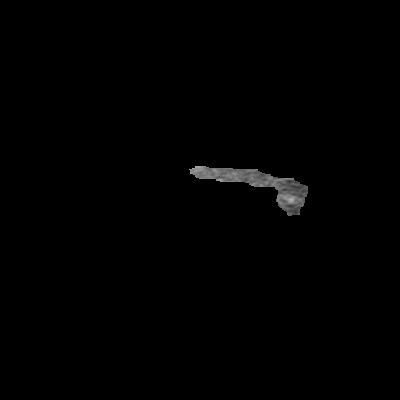

Supplement: S1 Data — (ZIP) [file pone.0212741.s001.zip › NotTear/36.bmp]

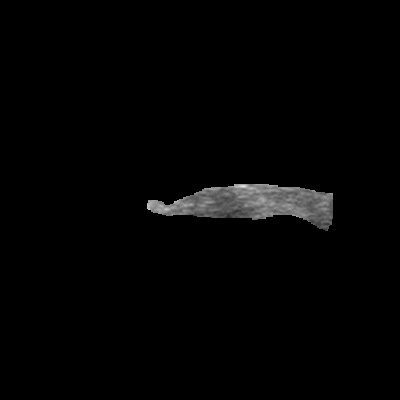

Supplement: S1 Data — (ZIP) [file pone.0212741.s001.zip › NotTear/37.bmp]

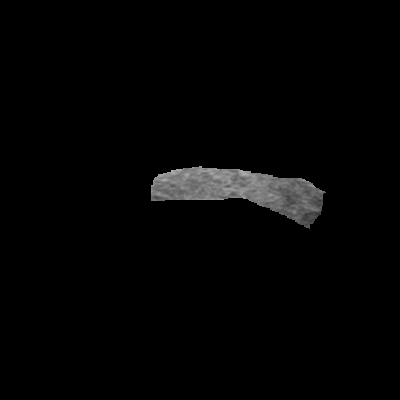

Supplement: S1 Data — (ZIP) [file pone.0212741.s001.zip › NotTear/38.bmp]

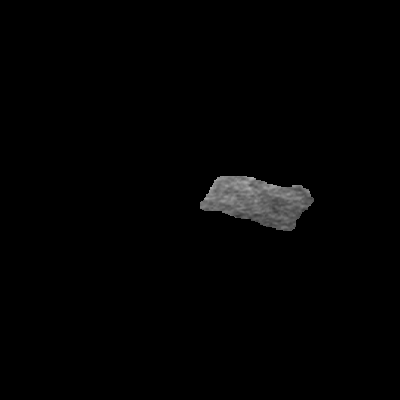

Supplement: S1 Data — (ZIP) [file pone.0212741.s001.zip › NotTear/39.bmp]

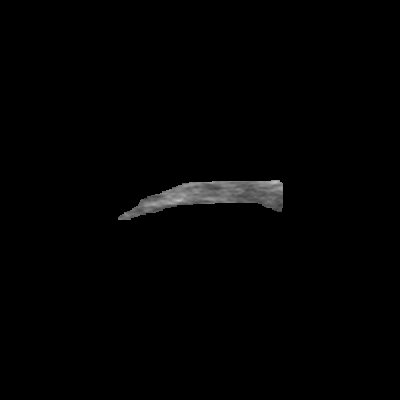

Supplement: S1 Data — (ZIP) [file pone.0212741.s001.zip › NotTear/4.bmp]

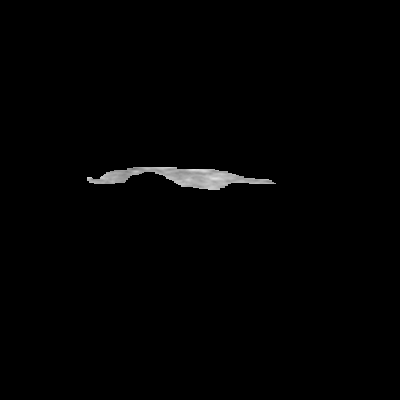

Supplement: S1 Data — (ZIP) [file pone.0212741.s001.zip › NotTear/40.bmp]

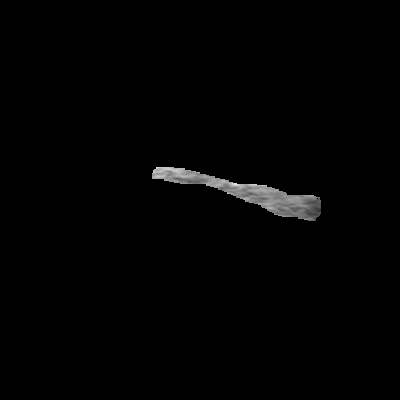

Supplement: S1 Data — (ZIP) [file pone.0212741.s001.zip › NotTear/41.bmp]

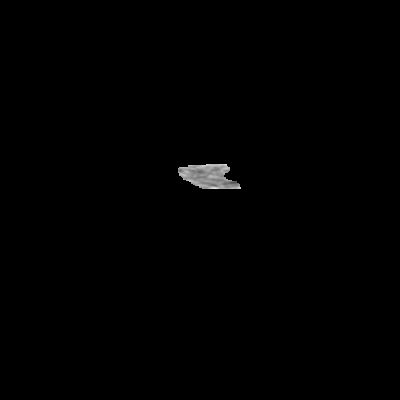

Supplement: S1 Data — (ZIP) [file pone.0212741.s001.zip › NotTear/42.bmp]

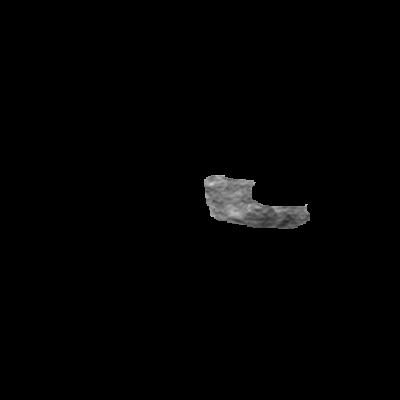

Supplement: S1 Data — (ZIP) [file pone.0212741.s001.zip › NotTear/43.bmp]

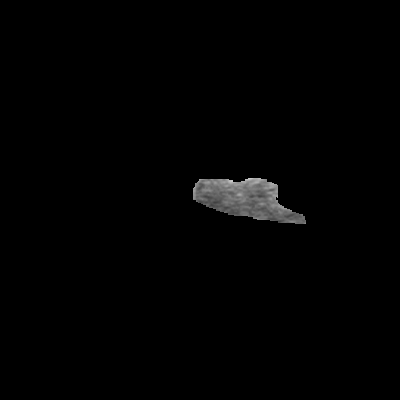

Supplement: S1 Data — (ZIP) [file pone.0212741.s001.zip › NotTear/44.bmp]

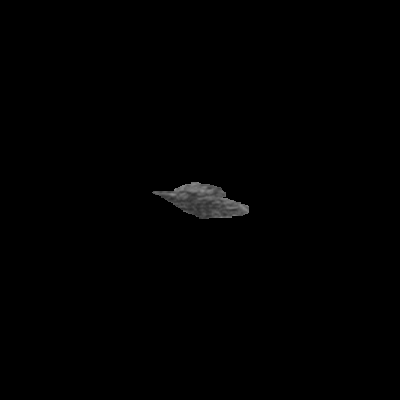

Supplement: S1 Data — (ZIP) [file pone.0212741.s001.zip › NotTear/45.bmp]

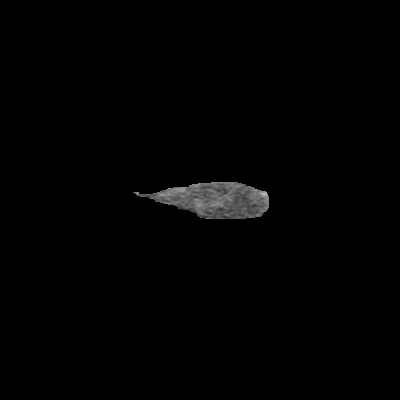

Supplement: S1 Data — (ZIP) [file pone.0212741.s001.zip › NotTear/46.bmp]

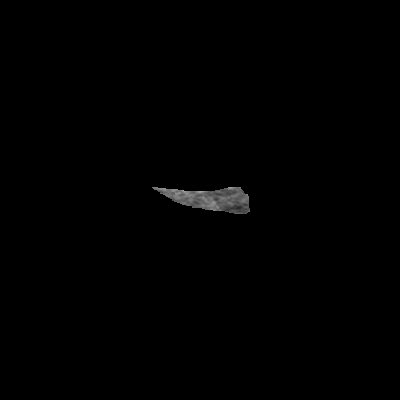

Supplement: S1 Data — (ZIP) [file pone.0212741.s001.zip › NotTear/47.bmp]

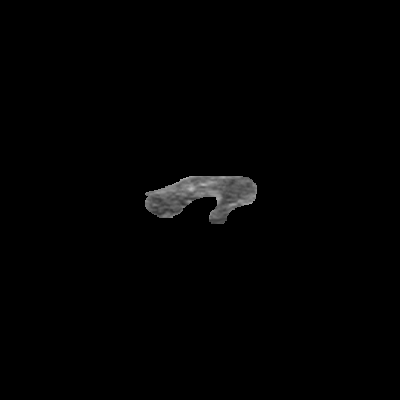

Supplement: S1 Data — (ZIP) [file pone.0212741.s001.zip › NotTear/48.bmp]

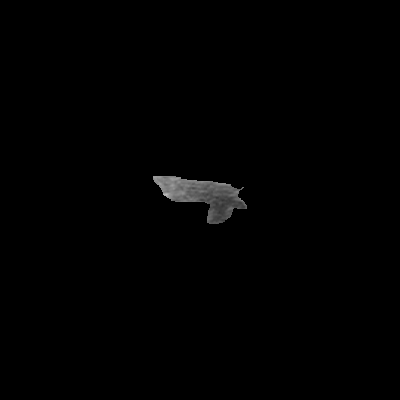

Supplement: S1 Data — (ZIP) [file pone.0212741.s001.zip › NotTear/49.bmp]

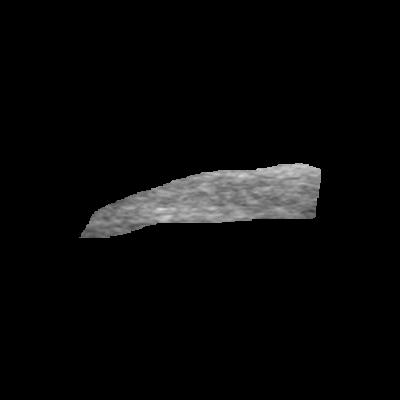

Supplement: S1 Data — (ZIP) [file pone.0212741.s001.zip › NotTear/5.bmp]

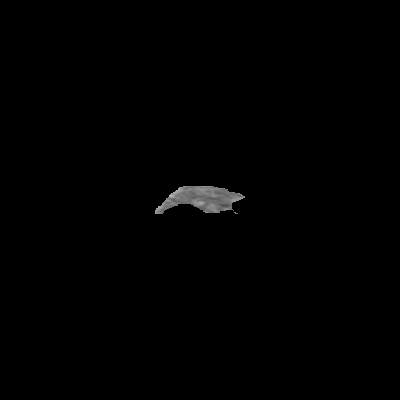

Supplement: S1 Data — (ZIP) [file pone.0212741.s001.zip › NotTear/50.bmp]

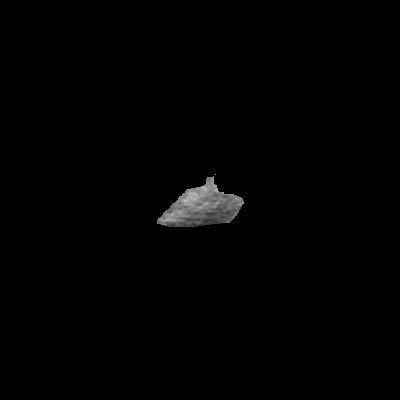

Supplement: S1 Data — (ZIP) [file pone.0212741.s001.zip › NotTear/51.bmp]

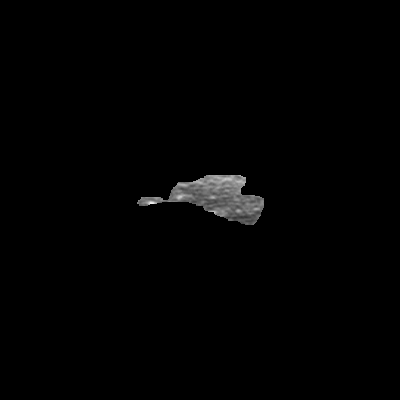

Supplement: S1 Data — (ZIP) [file pone.0212741.s001.zip › NotTear/52.bmp]

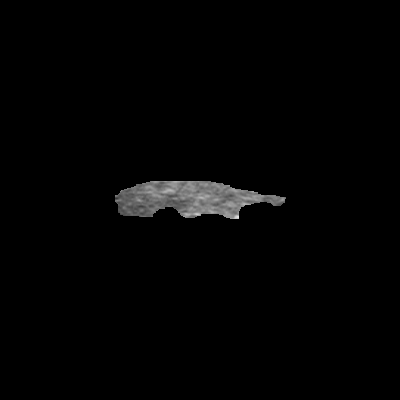

Supplement: S1 Data — (ZIP) [file pone.0212741.s001.zip › NotTear/53.bmp]

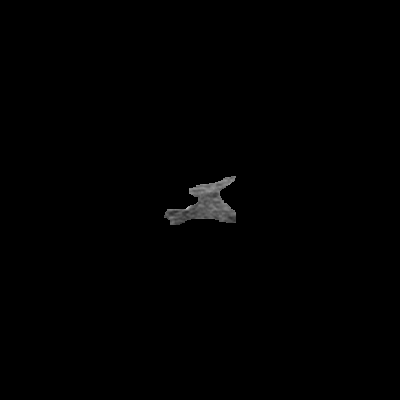

Supplement: S1 Data — (ZIP) [file pone.0212741.s001.zip › NotTear/54.bmp]

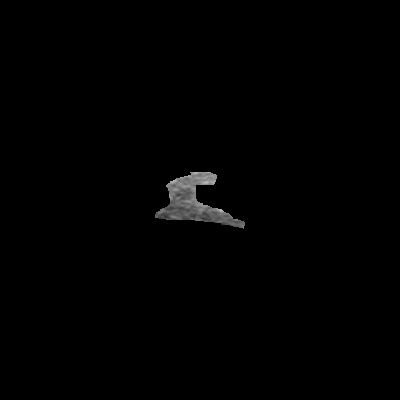

Supplement: S1 Data — (ZIP) [file pone.0212741.s001.zip › NotTear/55.bmp]

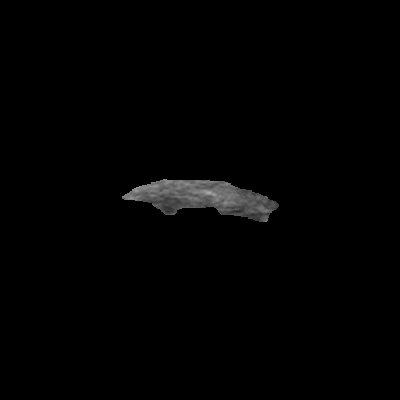

Supplement: S1 Data — (ZIP) [file pone.0212741.s001.zip › NotTear/56.bmp]

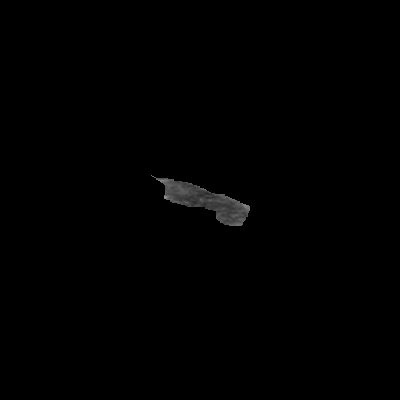

Supplement: S1 Data — (ZIP) [file pone.0212741.s001.zip › NotTear/57.bmp]

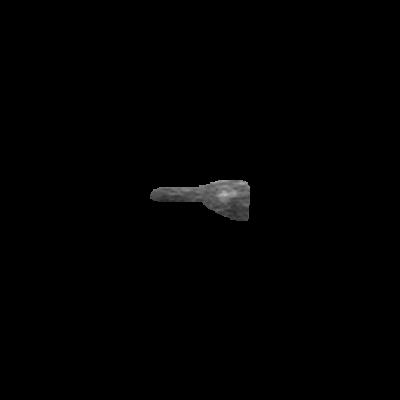

Supplement: S1 Data — (ZIP) [file pone.0212741.s001.zip › NotTear/58.bmp]

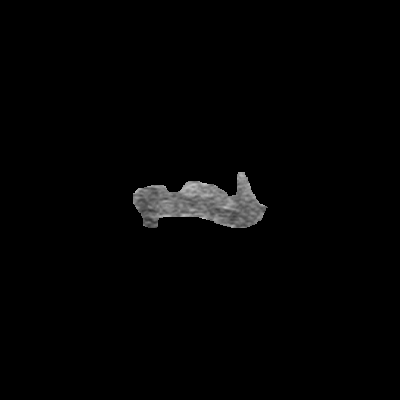

Supplement: S1 Data — (ZIP) [file pone.0212741.s001.zip › NotTear/59.bmp]

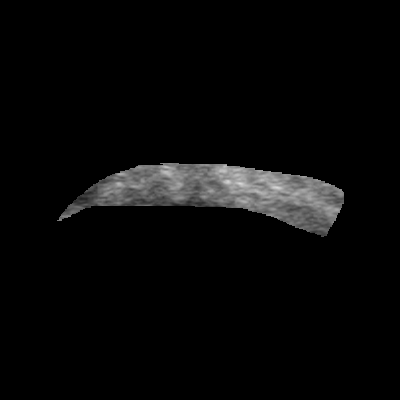

Supplement: S1 Data — (ZIP) [file pone.0212741.s001.zip › NotTear/6.bmp]

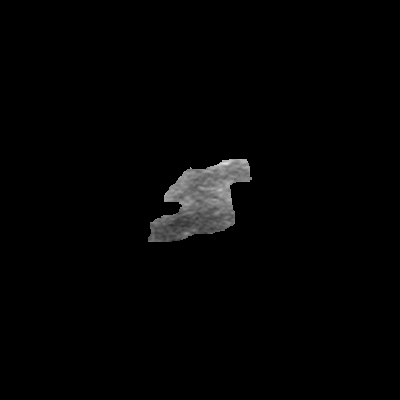

Supplement: S1 Data — (ZIP) [file pone.0212741.s001.zip › NotTear/60.bmp]

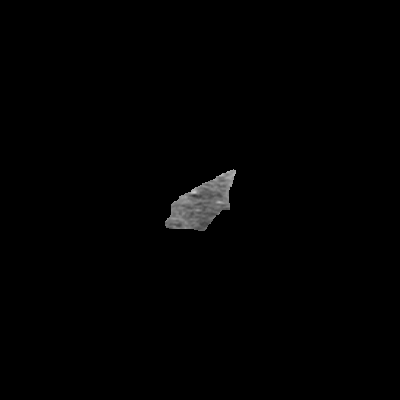

Supplement: S1 Data — (ZIP) [file pone.0212741.s001.zip › NotTear/61.bmp]

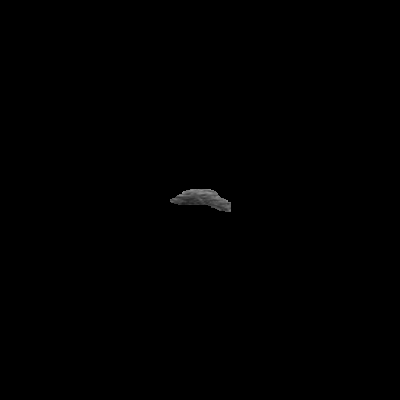

Supplement: S1 Data — (ZIP) [file pone.0212741.s001.zip › NotTear/62.bmp]

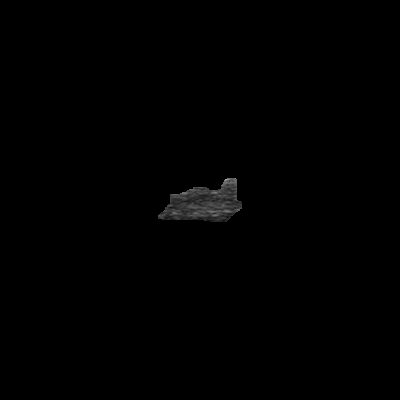

Supplement: S1 Data — (ZIP) [file pone.0212741.s001.zip › NotTear/63.bmp]

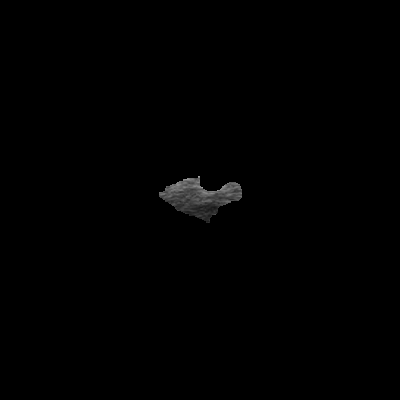

Supplement: S1 Data — (ZIP) [file pone.0212741.s001.zip › NotTear/64.bmp]

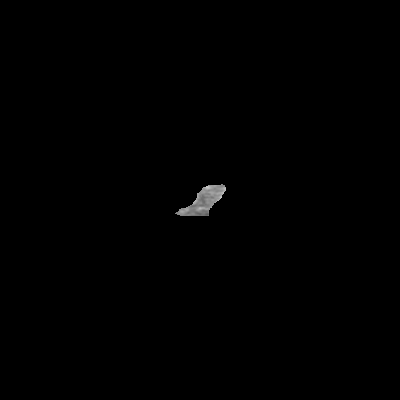

Supplement: S1 Data — (ZIP) [file pone.0212741.s001.zip › NotTear/65.bmp]

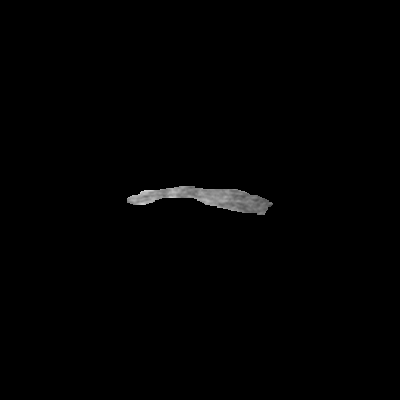

Supplement: S1 Data — (ZIP) [file pone.0212741.s001.zip › NotTear/66.bmp]

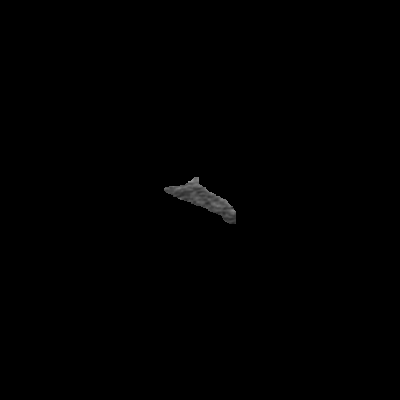

Supplement: S1 Data — (ZIP) [file pone.0212741.s001.zip › NotTear/67.bmp]

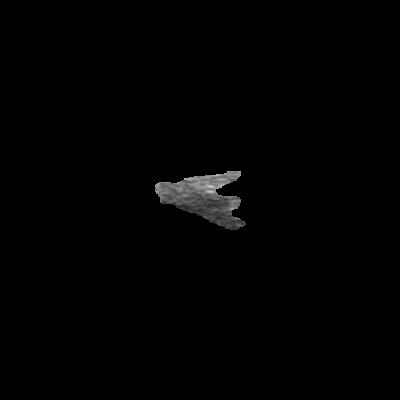

Supplement: S1 Data — (ZIP) [file pone.0212741.s001.zip › NotTear/68.bmp]

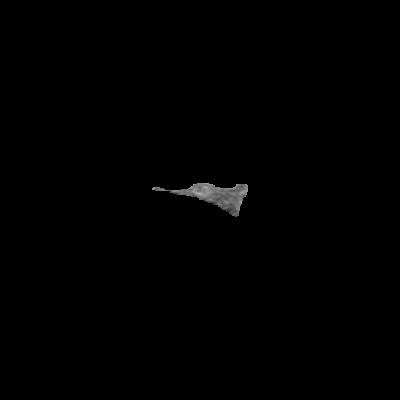

Supplement: S1 Data — (ZIP) [file pone.0212741.s001.zip › NotTear/69.bmp]

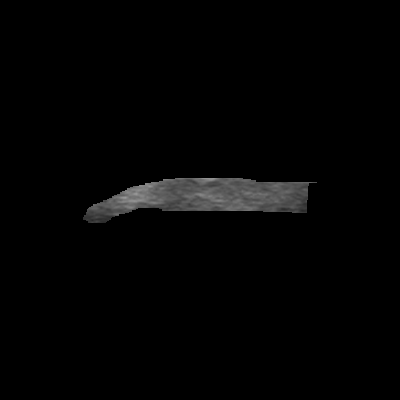

Supplement: S1 Data — (ZIP) [file pone.0212741.s001.zip › NotTear/7.bmp]

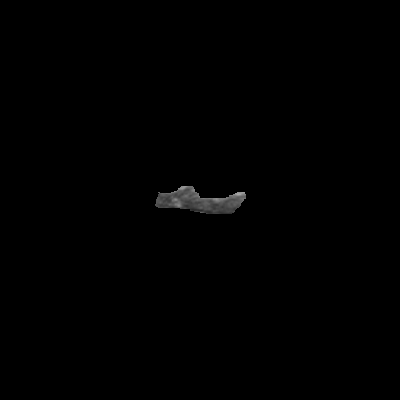

Supplement: S1 Data — (ZIP) [file pone.0212741.s001.zip › NotTear/70.bmp]

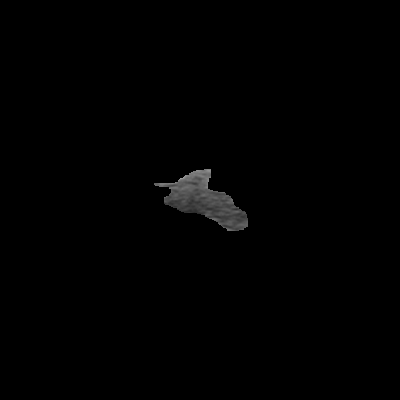

Supplement: S1 Data — (ZIP) [file pone.0212741.s001.zip › NotTear/71.bmp]

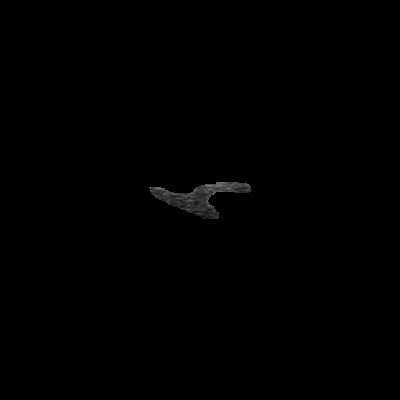

Supplement: S1 Data — (ZIP) [file pone.0212741.s001.zip › NotTear/72.bmp]

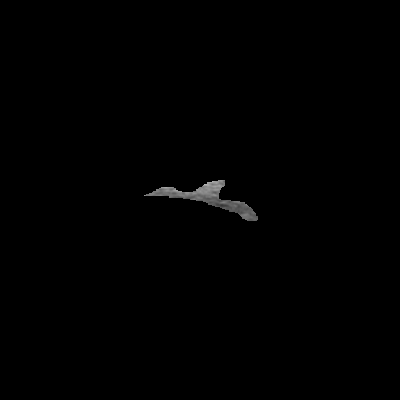

Supplement: S1 Data — (ZIP) [file pone.0212741.s001.zip › NotTear/73.bmp]

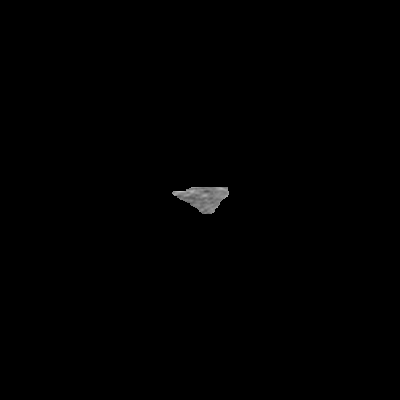

Supplement: S1 Data — (ZIP) [file pone.0212741.s001.zip › NotTear/74.bmp]

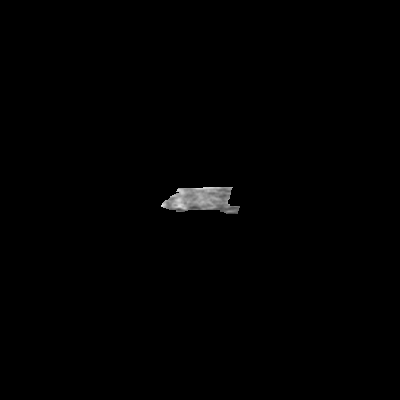

Supplement: S1 Data — (ZIP) [file pone.0212741.s001.zip › NotTear/75.bmp]

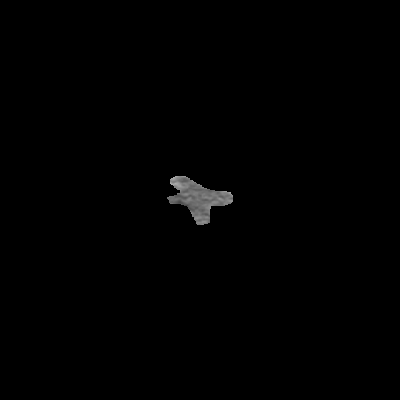

Supplement: S1 Data — (ZIP) [file pone.0212741.s001.zip › NotTear/76.bmp]

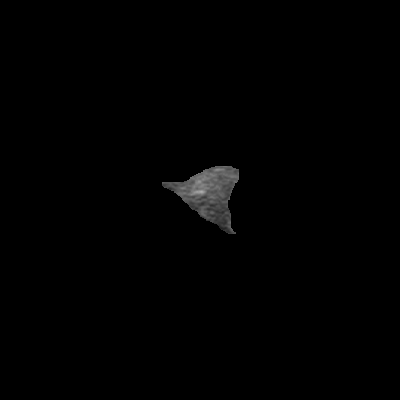

Supplement: S1 Data — (ZIP) [file pone.0212741.s001.zip › NotTear/77.bmp]

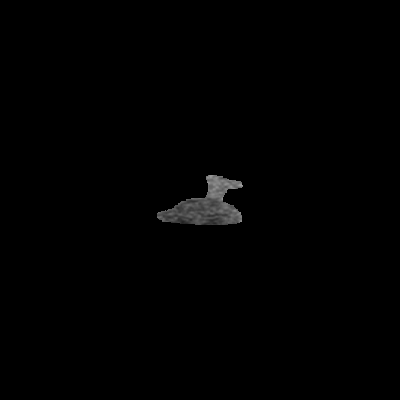

Supplement: S1 Data — (ZIP) [file pone.0212741.s001.zip › NotTear/78.bmp]

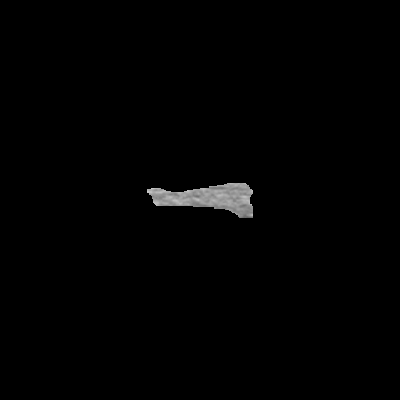

Supplement: S1 Data — (ZIP) [file pone.0212741.s001.zip › NotTear/79.bmp]

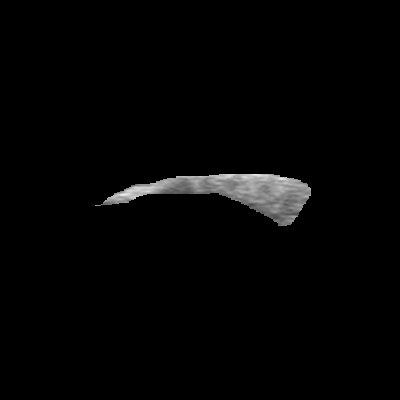

Supplement: S1 Data — (ZIP) [file pone.0212741.s001.zip › NotTear/8.bmp]

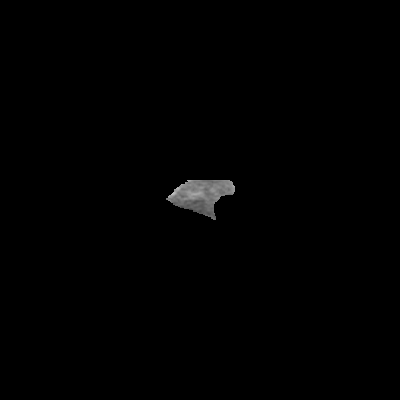

Supplement: S1 Data — (ZIP) [file pone.0212741.s001.zip › NotTear/80.bmp]

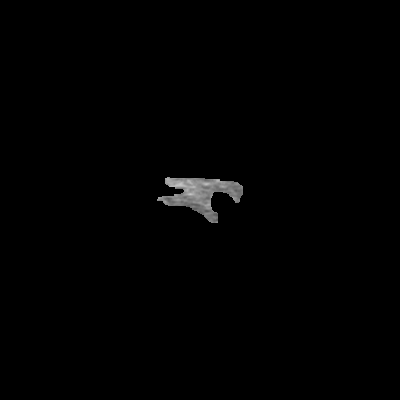

Supplement: S1 Data — (ZIP) [file pone.0212741.s001.zip › NotTear/81.bmp]

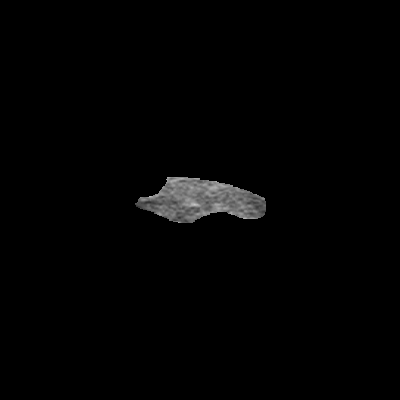

Supplement: S1 Data — (ZIP) [file pone.0212741.s001.zip › NotTear/82.bmp]

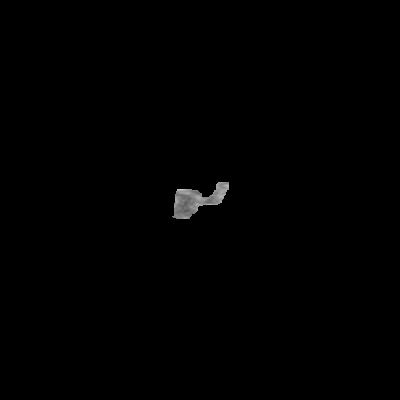

Supplement: S1 Data — (ZIP) [file pone.0212741.s001.zip › NotTear/83.bmp]

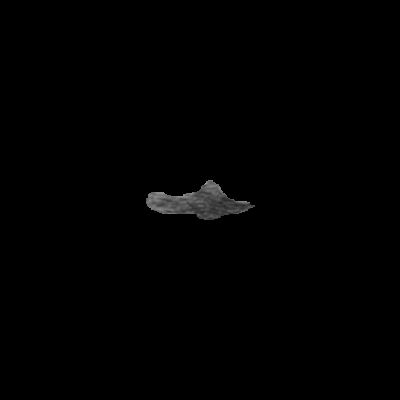

Supplement: S1 Data — (ZIP) [file pone.0212741.s001.zip › NotTear/84.bmp]

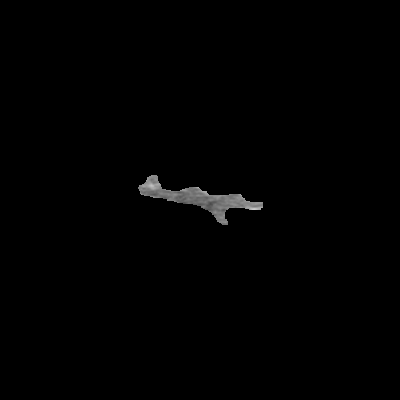

Supplement: S1 Data — (ZIP) [file pone.0212741.s001.zip › NotTear/85.bmp]

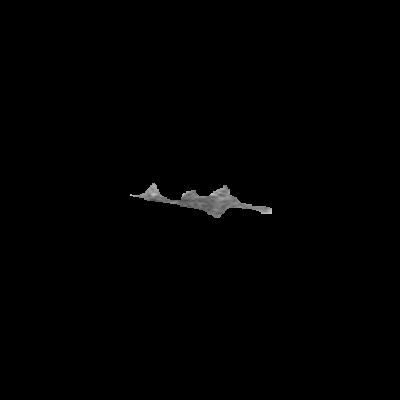

Supplement: S1 Data — (ZIP) [file pone.0212741.s001.zip › NotTear/86.bmp]

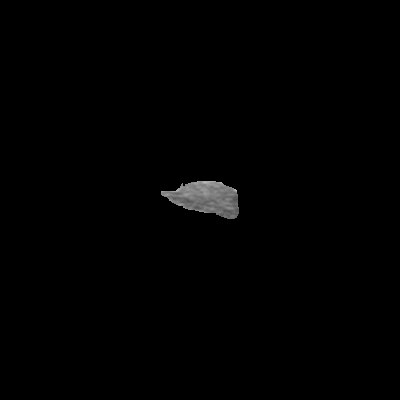

Supplement: S1 Data — (ZIP) [file pone.0212741.s001.zip › NotTear/87.bmp]

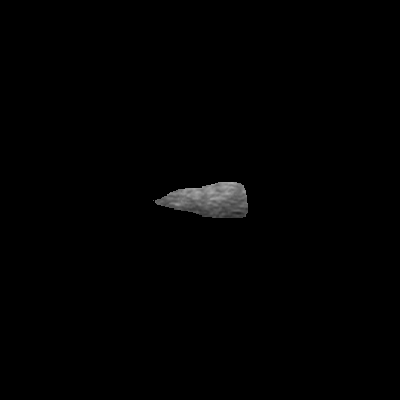

Supplement: S1 Data — (ZIP) [file pone.0212741.s001.zip › NotTear/88.bmp]

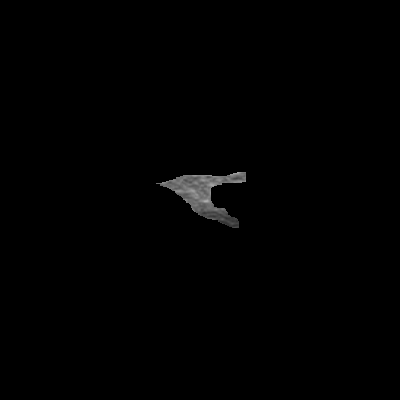

Supplement: S1 Data — (ZIP) [file pone.0212741.s001.zip › NotTear/89.bmp]

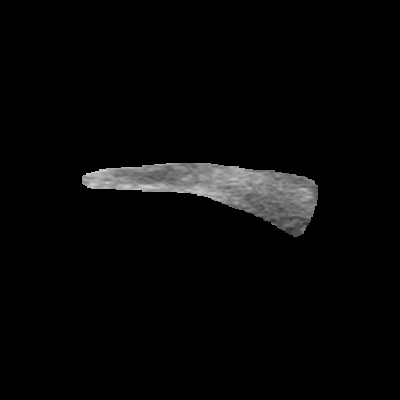

Supplement: S1 Data — (ZIP) [file pone.0212741.s001.zip › NotTear/9.bmp]

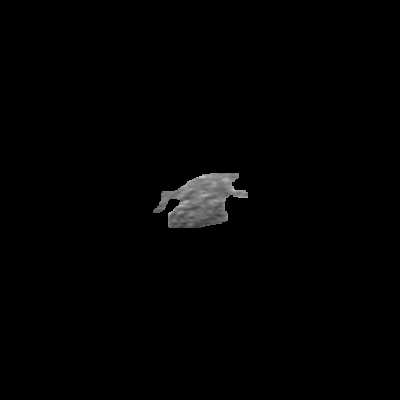

Supplement: S1 Data — (ZIP) [file pone.0212741.s001.zip › NotTear/90.bmp]

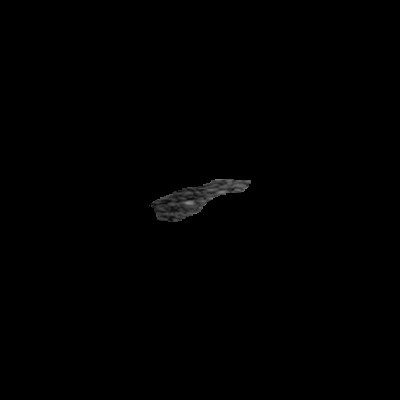

Supplement: S1 Data — (ZIP) [file pone.0212741.s001.zip › Tear/1.bmp]

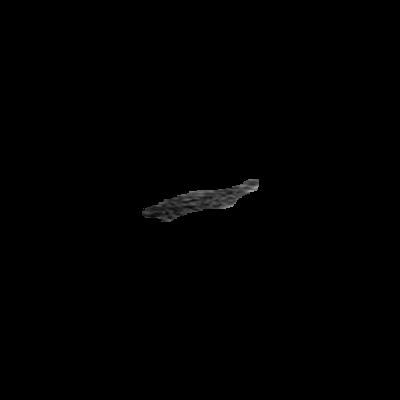

Supplement: S1 Data — (ZIP) [file pone.0212741.s001.zip › Tear/10.bmp]

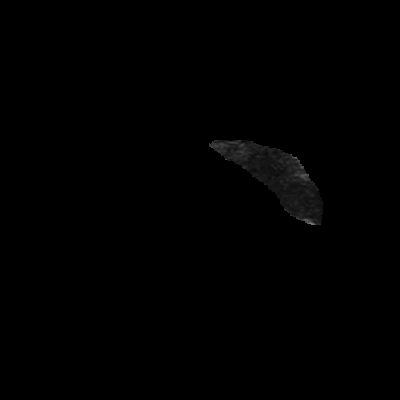

Supplement: S1 Data — (ZIP) [file pone.0212741.s001.zip › Tear/100.bmp]

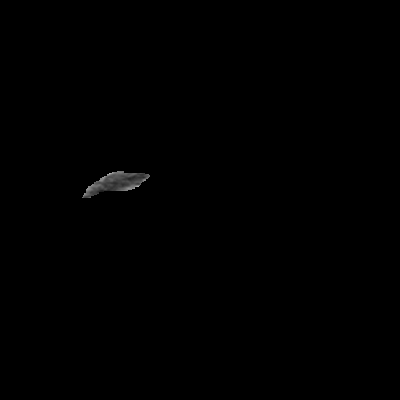

Supplement: S1 Data — (ZIP) [file pone.0212741.s001.zip › Tear/101.bmp]

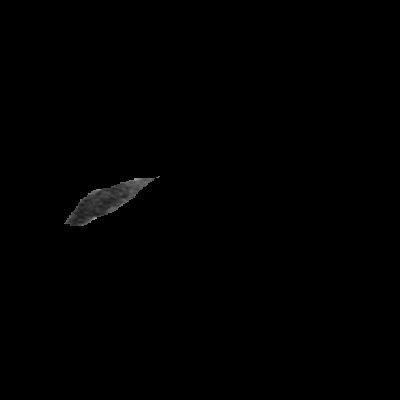

Supplement: S1 Data — (ZIP) [file pone.0212741.s001.zip › Tear/102.bmp]

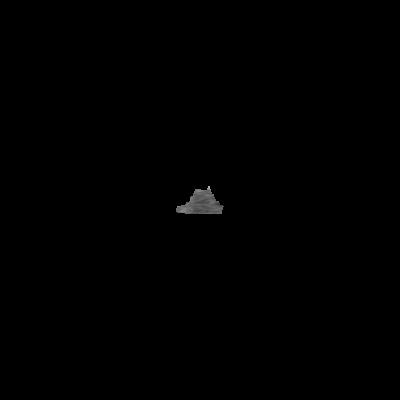

Supplement: S1 Data — (ZIP) [file pone.0212741.s001.zip › Tear/11.bmp]

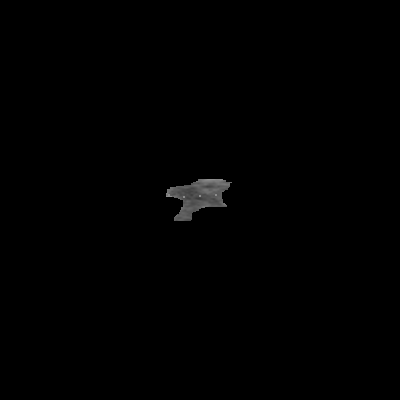

Supplement: S1 Data — (ZIP) [file pone.0212741.s001.zip › Tear/12.bmp]

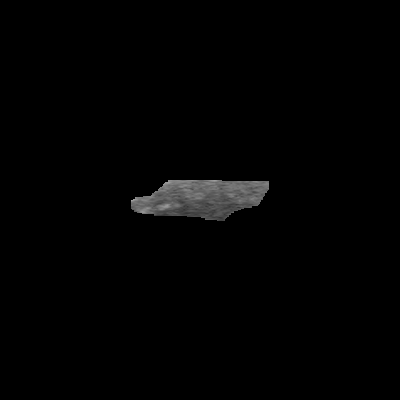

Supplement: S1 Data — (ZIP) [file pone.0212741.s001.zip › Tear/13.bmp]

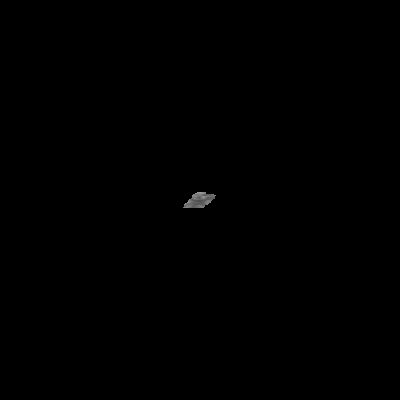

Supplement: S1 Data — (ZIP) [file pone.0212741.s001.zip › Tear/14.bmp]

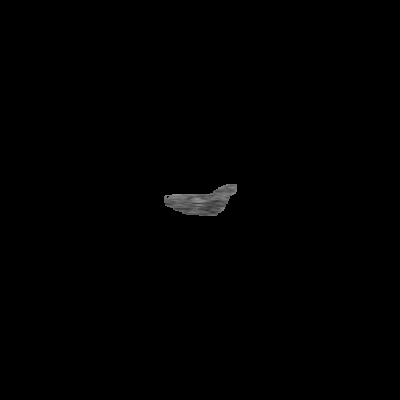

Supplement: S1 Data — (ZIP) [file pone.0212741.s001.zip › Tear/15.bmp]

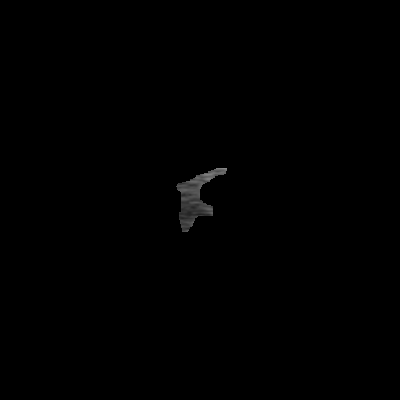

Supplement: S1 Data — (ZIP) [file pone.0212741.s001.zip › Tear/16.bmp]
